# Rationally introducing non-canonical amino acids to enhance catalytic activity of LmrR for Henry reaction

**DOI:** 10.1186/s40643-024-00744-w

**Published:** 2024-02-29

**Authors:** Lan Wang, Mengting Zhang, Haidong Teng, Zhe Wang, Shulin Wang, Pengcheng Li, Jianping Wu, Lirong Yang, Gang Xu

**Affiliations:** 1 Institute of Bioengineering, College of Chemical and Biological Engineering, Zhejiang University, Hangzhou, 310027 Zhejiang China; 2 Huadong Medicine Co., Ltd, Hangzhou, 310011 Zhejiang China

**Keywords:** Henry reaction, Catalytic activity, Rational design, Non-canonical amino acid, Molecular dynamics simulations

## Abstract

The use of enzymes to catalyze Henry reaction has advantages of mild reaction conditions and low contamination, but low enzyme activity of promiscuous catalysis limits its application. Here, rational design was first performed to identify the key amino acid residues in Henry reaction catalyzed by Lactococcal multidrug resistance Regulator (LmrR). Further, non-canonical amino acids were introduced into LmrR, successfully obtaining variants that enhanced the catalytic activity of LmrR. The best variant, V15CNF, showed a 184% increase in enzyme activity compared to the wild type, and was 1.92 times more effective than the optimal natural amino acid variant, V15F. Additionally, this variant had a broad substrate spectrum, capable of catalyzing reactions between various aromatic aldehydes and nitromethane, with product yielded ranging from 55 to 99%. This study improved enzymatic catalytic activity by enhancing affinity between the enzyme and substrates, while breaking limited types of natural amino acid residues by introducing non-canonical amino acids into the enzyme, providing strategies for molecular modifications.

**Graphical Abstract:**

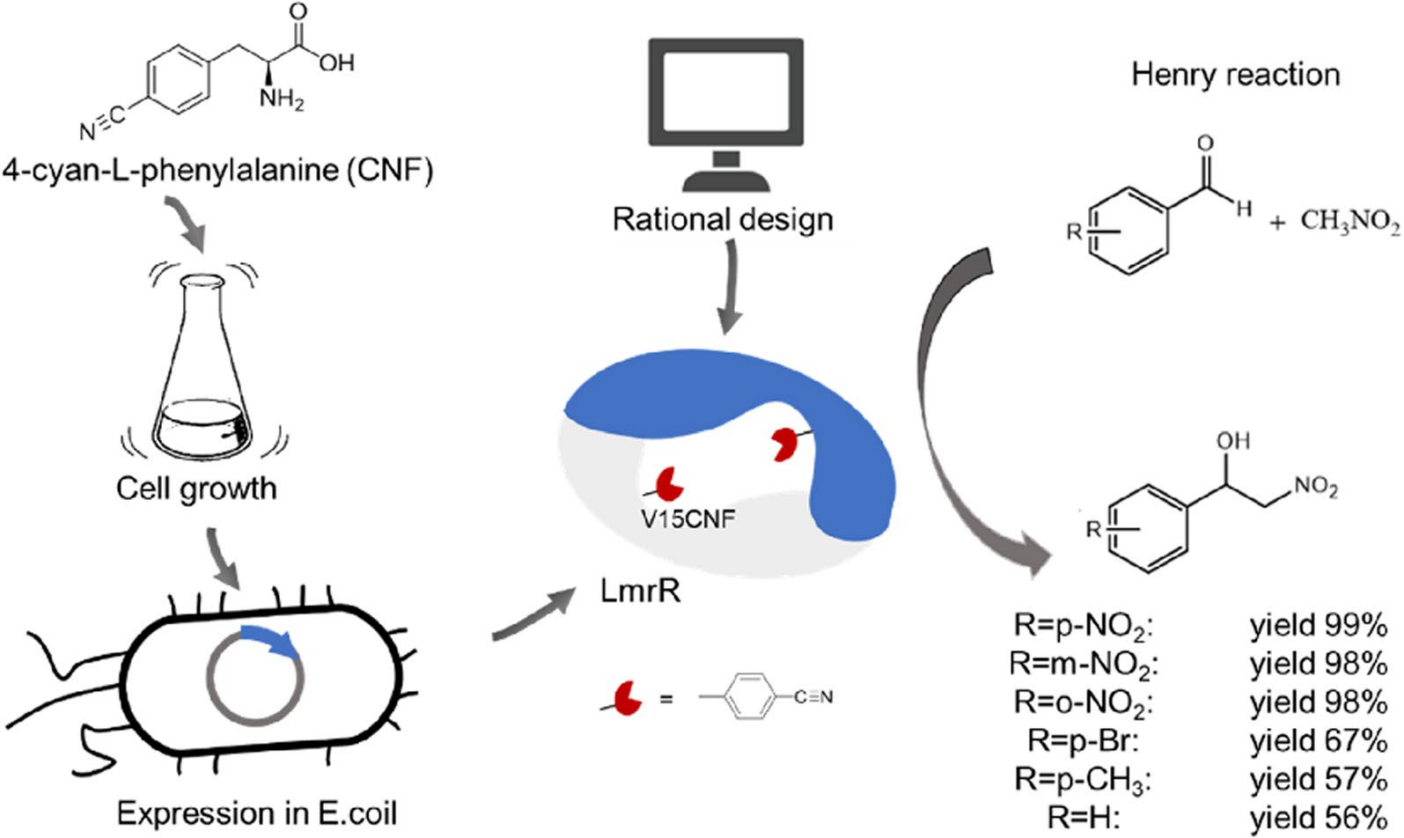

**Supplementary Information:**

The online version contains supplementary material available at 10.1186/s40643-024-00744-w.

## Introduction

Henry reaction is commonly used for carbon chain elongation and has excellent atom economy in the construction of carbon–carbon bonds (Luzzio 2001). In addition, the reaction products β-nitroalcohols are important organic synthetic intermediates that can be used to prepare various chemical products and active pharmaceuticals, such as insecticides, antibiotics, amino alcohols, and ephedrine (Xia et al. 2021; Sinéad et al. 2012). Many organic bases, inorganic bases, or quaternary ammonium salts have been used to catalyze this reaction (Simoni et al. 1997; Marcelli et al. 2006), but these catalysts generally have disadvantages such as complex preparation processes, significant environmental pollution, high toxicity, and multiple side reactions (Anastas et al. 2010). Therefore, there is an urgent need to develop cleaner, greener, and more efficient catalytic systems.

Biocatalysts are natural catalysts that have the advantages of mild reaction conditions, low pollution, and enzyme immobilization for multiple uses (Sánchez et al. 2018). Using biocatalysts to catalyze Henry reaction is in line with the development direction of green catalysis and has great potential for development. Currently, various types of enzymes have been reported to be used for hybrid catalysis in Henry reaction, including glutaminase, lipase, hydroxynitrile lyase, and acyl transferase (Tang et al. 2010; Kühbeck et al. 2013; Yu et al. 2020; Fuhshuku and Asano 2011). Regarding the reactions between nitrobenzaldehyde and nitromethane, Table 1 presents the catalytic performance of various enzymes in current research. The related reports indicate that current optimization of enzymes for Henry reaction is mainly achieved through the optimization of reaction conditions and chemical or immobilization modifications. The design of subsequent enzyme modification schemes can be more effectively employed to enhance enzyme activity, and besides, it allows for the analysis of enzyme catalytic mechanisms.

**Table 1:** Different types of enzymes used to catalyze Henry reaction

Entry	Catalyst	Optimization	Time /h	Yield /%	Ref.
1	LmrR	Genetic modification	8	99	This study
2	porcine skin type-A (PSTA)	Gelatin-mediated	6	70	(Kühbeck et al. 2013)
3	Lipase from Rhizopus niveus (RNL)	Chemical modification	24	99	(Yu et al. 2020)
4	Transglutaminase	Immobilization modification and reaction condition optimization	48	96	(Tang et al. 2010)

For enzyme-catalyzed Henry reaction, we started by selecting enzymes based on the binding of substrates, giving rise to a high effective concentration of substrates, was a very important contributor to the enzymatic rate enhancement of bimolecular reactions by removing the entropic cost from the rate-limiting step. Hence the design starts by selecting a protein scaffold that provides a suitable binding pocket.

The Lactococcal multidrug resistance Regulator (LmrR) is associated with the drug resistance of Lactobacillus lactis (Van Der Berg et al. 2015; Agustiandari et al. 2011, 2008). LmrR contains a large hydrophobic binding pocket, providing generic binding interactions. Therefore, LmrR is inherently promiscuous in binding of hydrophpbic drugs. The reason why this is attractive is because organic substrates in Henry reaction, which are hydrophobic, will like to bind here (Roelfes 2019). The hydrophobic binding pocket is highly flexible and readily adapts to the bound guest molecule (Takeuchi et al. 2014). Roelfes et al. designed an artificial enzyme using LmrR, simultaneously introducing unnatural p-aminophenylalanine residue and a phenanthroline copper complex into the hydrophobic cavity. These components synergistically catalysed Michael addition reaction of two substrates (Zhou and Roelfes 2020). This means that LmrR allows for the introduction of bulky non-canonical amino acids and cofactors and provides extra space for substrate binding (Roelfes 2019; Zhou and Roelfes 2020; Leveson-Gower et al. 2022). LmrR is often used as a protein scaffold for artificial enzymes (Bos et al. 2012, 2013b, 2015; Drienovská et al. 2017; Leveson-Gower et al. 2022, 2021; Villarino et al. 2018). The commonly employed method is introducing an unnatural catalytic transition-metal complex that already has some basal activity in the reaction of interest. For acid–base catalyzed reactions like the Henry reaction, the method involves ensuring the substrate's binding with the enzyme. The residues of the protein scaffold can provide an active catalytic environment (Leveson-Gower et al. 2021). It is known that many residues of LmrR have been identified to facilitate catalysis. Among them, D100 in LmrR, serves as a general base in the addition of water to enones, activating the nucleophilic water molecule (Bos et al. 2013a). In addition, the crystal structure of LmrR is well-defined and has strong structural designability (Zhou and Roelfes 2020), making it easy for subsequent modification and exploration of catalytic mechanisms. Therefore, this study focused on LmrR-catalyzed Henry reaction and molecular modification of LmrR.

In this study, Henry reaction between p-nitrobenzaldehyde and nitromethane was chosen as the model reaction. Computer-aided calculations (Teng et al. 2023) were used to predict key binding sites for the catalytic Henry reaction, and site-directed mutagenesis was performed to validate effects. Based on the above process, we hypothesized mechanism of the LmrR-catalyzed Henry reaction. Besides, introduction of non-canonical amino acids into the enzyme molecule was used to further enhance the enzyme's catalytic activity. Molecular dynamics simulations were also employed to explain the reasons behind the increased enzyme activity.

## Results and discussion

### Selection of candidate amino acid residues

**Fig. 1:**
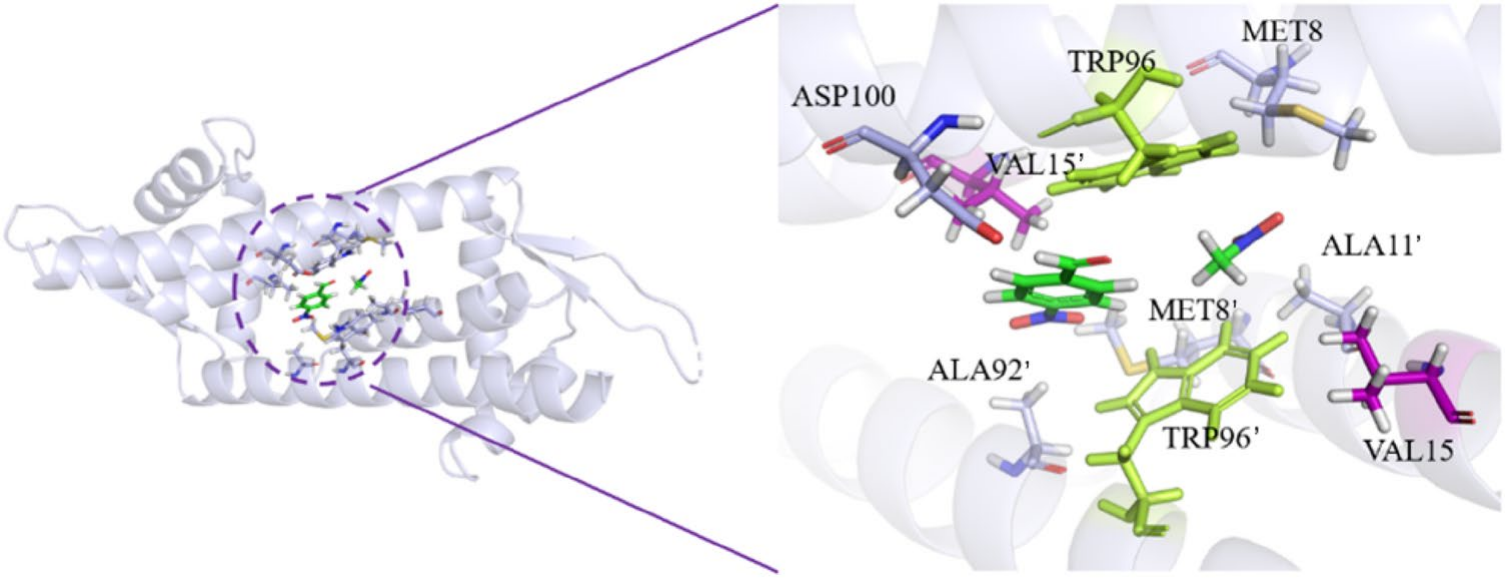
Molecular docking results of LmrR

LmrR is a homodimeric protein with a large hydrophobic pocket in its spatial structure (to distinguish amino acids originating from two identical monomers, the amino acid labels from one monomer are annotated with a “'”). Two substrates (p-nitrobenzaldehyde and nitromethane) were gathered inside the LmrR hydrophobic pocket, with 9 amino acid residues within 5 Å range of the two substrates, namely Asp100, Val15, Trp96, Met8, Ala92', Trp96', Met8', Ala11' and Val15' (Fig. 1). Among them, Trp96 and Trp96' are important residues that maintain the dimeric structure. Trp96/Trp96' are one of the residues involved in forming the hydrophobic dimer interface (Madoori et al. 2008). Trp96/Trp96' are located at the center of the dimeric pore, with the indole rings oriented in a parallel direction, engaging in π–π interactions, thereby stabilizing the dimer and expanding the hydrophobic free volume of the cavity (Villarino et al. 2020; Ferrara et al. 2022).

Site-directed mutations of protein amino acids can be used for enzyme design, but blindly selecting amino acid residues for enzyme promiscuous catalysis will lead to low efficiency. Therefore, in this study, virtual amino acid mutations combined with molecular docking results were used to determine the best amino acid mutation method (Leveson-Gower et al. 2021).

First, alanine scan was performed on the amino acid residues within 10 Å of two ligand small molecules after docking, with a total of 65 calculation results. The mutation energies were ranked from largest to smallest, and the top 10 results were shown in Table 2. It could be seen that the mutations of Trp96, Trp96', and Val15' to alanine had a mutation energy greater than 0.5, which led to a decrease in the affinity between the enzyme and substrates. Therefore, it could be inferred that the 96th and 15th positions were key amino acid residues for LmrR catalyzing Henry reaction.

**Table 2:** Alanine scanning

Mutation	Mutation Energy^a^ (kcal/mol)	Effect
TRP96 > ALA	2.43	Destabilizing
TRP96' > ALA	1.51	Destabilizing
VAL15' > ALA	0.67	Destabilizing
ASP100 > ALA	0.41	Neutral
MET8 > ALA	0.37	Neutral
MET8' > ALA	0.23	Neutral
ILE103 > ALA	0.14	Neutral
VAL99 > ALA	0.09	Neutral
VAL15 > ALA	0.09	Neutral
PRO5' > ALA	0.05	Neutral

^a^When the mutation energy is greater than 0.5, the Effect is destabilizing, indicating that the mutation will lead to a decrease in the affinity between the enzyme and substrates; when the mutation energy is between − 0.5 ~  + 0.5, the Effect is neutral, indicating that the mutation has no effect on the affinity between the enzyme and substrates

**Fig. 2:**
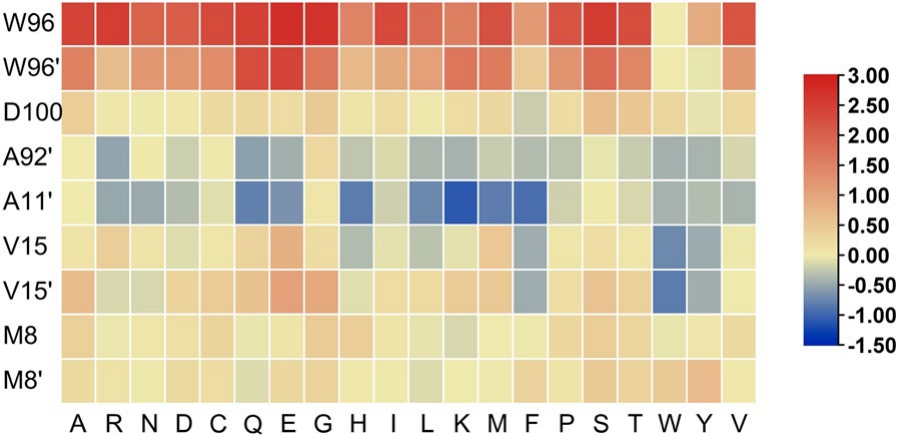
Heat map of ∆∆G between variants and wild type of LmrR (red: ∆∆G > 0; yellow: ∆∆G = 0; blue: ∆∆G < 0)

In addition to the aforementioned key amino acid residues, to avoid missing potentially positive positions, six extra active site residues including Ala92ʹ, Ala11ʹ, Val15ʹ, Asp100, Met8 and Met8' were also selected as keys amino acids. Random mutation at all these residues would still be a challenge for library construction and screening. Herein, we successively employed the in silico screening and site-directed mutagenesis method to shrink the amount of amino acid residues and identify the candidates with a significant influence on enzymatic activity (Zheng et al. 2021). In silico screening was carried out using the protocol Calculate Mutation Energy (Binding) of Discovery Studio 4.0 (DS) and the abovementioned amino acid residues were mutated to other 19 amino acid residues (Zheng et al. 2020; Petukh et al. 2016; Zong et al. 2015). A total of 171 computational results were obtained, and Fig. 2 showed the heatmap of the saturation mutagenesis scan results. It could be observed that when residues W96 and W96ʹ were mutated to any of the other 19 amino acids, the colors were shown in red, indicating that compared to the wild-type, mutations at residues W96 and W96ʹ increased the binding free energy and led to a decrease in enzyme-substrates affinity, therefore, these sites were not suitable for mutations. When residues D100, M8 and M8ʹ were mutated to other 19 amino acids, the colors were shown in yellow, indicating that the binding free energy of their variants changed insignificantly as compared to the wild-type, therefore, these sites were also not suitable for mutations. However, when residues A11ʹ, V15, V15ʹ and A92ʹ were mutated to other 19 amino acids, the heatmaps contained blue, indicating that these variants led to a decrease in binding free energy and an increase in enzyme-substrates affinity and interaction strength, therefore, these sites were suitable for enhancing enzyme activity through mutations.

After identifying the mutation sites through heatmaps, further determination of the exact mutation types was performed. The mutation energies from the saturated mutation results were arranged in ascending order (blue from deep to shallow in Fig. 2), and the top twenty results were listed in Table 3. The mutation energies of the first 11 rows, A11ʹK, A11ʹF, A11ʹH, V15ʹW, A11ʹM, A11ʹQ, A11ʹL, V15W, A11ʹE, A92ʹQ, and A92ʹR were less than -0.5, with the effect being stabilizing. This indicated that these mutations would enhance the interaction between the enzyme and substrates, thereby making these 11 mutations the best options for virtual screening. The mutation energies of the last 9 rows, A11ʹR, A11ʹN, V15Y, V15F, V15ʹF, V15ʹY, A92ʹE, A92ʹW, and A11ʹW were between − 0.5 and − 0.4, with the effect being neutral. This suggested that there might be mutation sites among these points that could improve the catalytic performance. To avoid missing potentially positive variants, the study also included them in the mutation screening library. Additionally, LmrR is a dimer, and a mutation at one site would cause both subunits in the dimer to mutate. Therefore, based on the saturated mutation scan, computer-aided design eventually identified 17 variants that could potentially increase enzyme activity.

**Table 3:** Saturated mutation scanning

Mutation	Mutation Energy^a^ (kcal/mol)	Effect
A11ʹK	− 1.09	Stabilizing
A11ʹF	− 0.92	Stabilizing
A11ʹH	− 0.82	Stabilizing
V15ʹW	− 0.82	Stabilizing
A11ʹM	− 0.81	Stabilizing
A11ʹQ	− 0.78	Stabilizing
A11ʹL	− 0.73	Stabilizing
V15W	− 0.72	Stabilizing
A11ʹE	− 0.65	Stabilizing
A92ʹQ	− 0.55	Stabilizing
A92ʹR	− 0.52	Stabilizing
A11ʹR	− 0.50	Neutral
A11ʹN	− 0.48	Neutral
V15Y	− 0.47	Neutral
V15F	− 0.45	Neutral
V15ʹF	− 0.45	Neutral
V15ʹY	− 0.44	Neutral
A92ʹE	− 0.43	Neutral
A92ʹW	− 0.42	Neutral
A11ʹW	− 0.41	Neutral

^a^When the mutation energy is less than − 0.5, the Effect is stabilizing, indicating that the mutation will lead to an increase in the affinity between the enzyme and substrates; when the mutation energy is between − 0.5 ~  + 0.5, the Effect is neutral, indicating that the mutation has no effect on the affinity between the enzyme and substrates

### Screening of the variant library

**Fig. 3:**
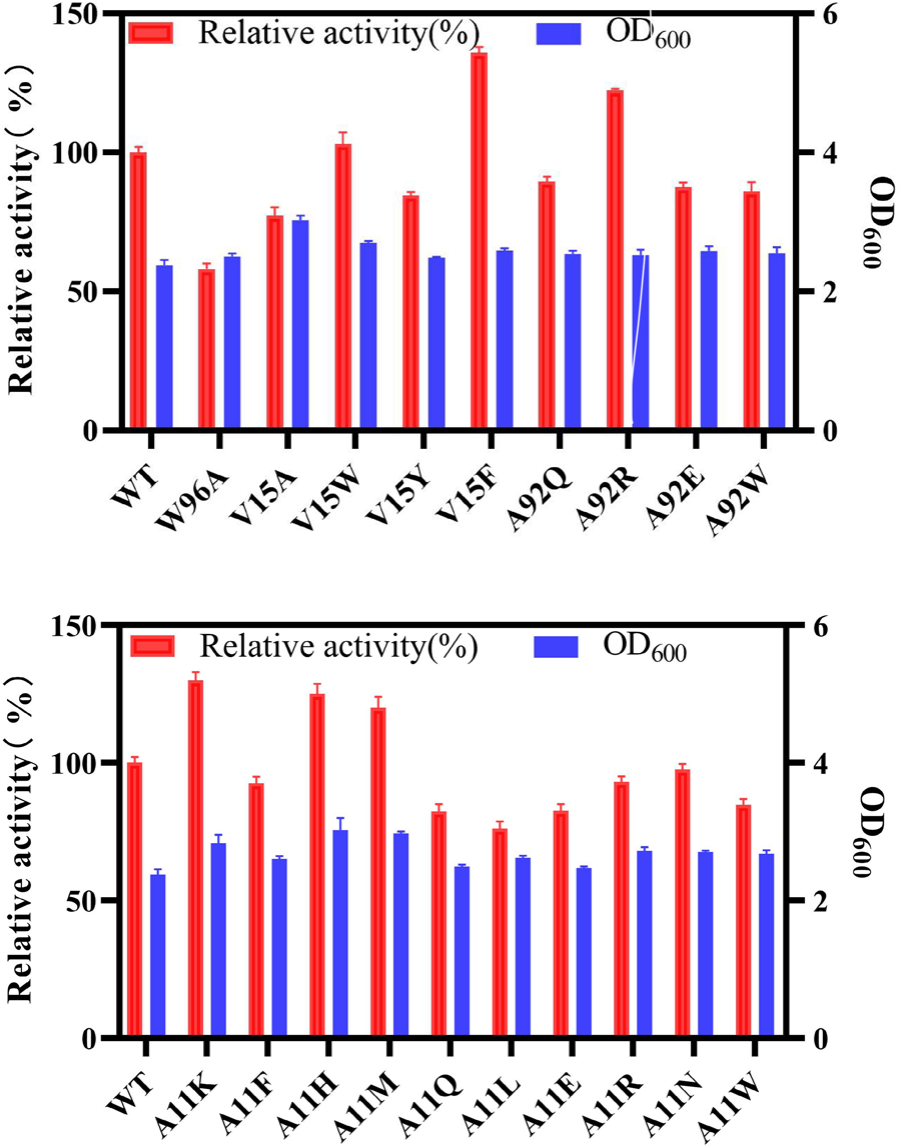
Comparison of enzyme activity between wild type and variants of LmrR

The key residues amino acid V15 and W96 obtained from alanine scanning were mutated to alanine. In addition, 17 saturation mutations were performed at specific sites, including A11K, A11F, A11H, A11M, A11Q, A11L, A11E, A11R, A11N, A11W, V15W, V15Y, V15F, A92Q, A92R, A92E, and A92W (the protein is a homodimer, all changes to the protein occur twice. Therefore, amino acid labeling is no longer being distinguished). Enzyme assays were carried out using crude enzyme extracts, with the enzyme activity of wild-type LmrR set at 100%, and relative enzyme activity of other variants were calculated. The results were summarized in Fig. 3. It showed that the enzyme activity of variants W96A and V15A decreased by 58% and 77%, respectively, indicating that W96 and V15 were key amino acid residues for catalyzing Henry reaction of LmrR, which was consistent with the results of the alanine scanning. Among the 17 point mutations, V15F, A92R, A11K, A11H, and A11M had significant increases in enzyme activity, with relative enzyme activities of 136%, 122%, 130%, 125%, and 120%, respectively.

### Analysis of LmrR catalysis mechanism

**Fig. 4:**
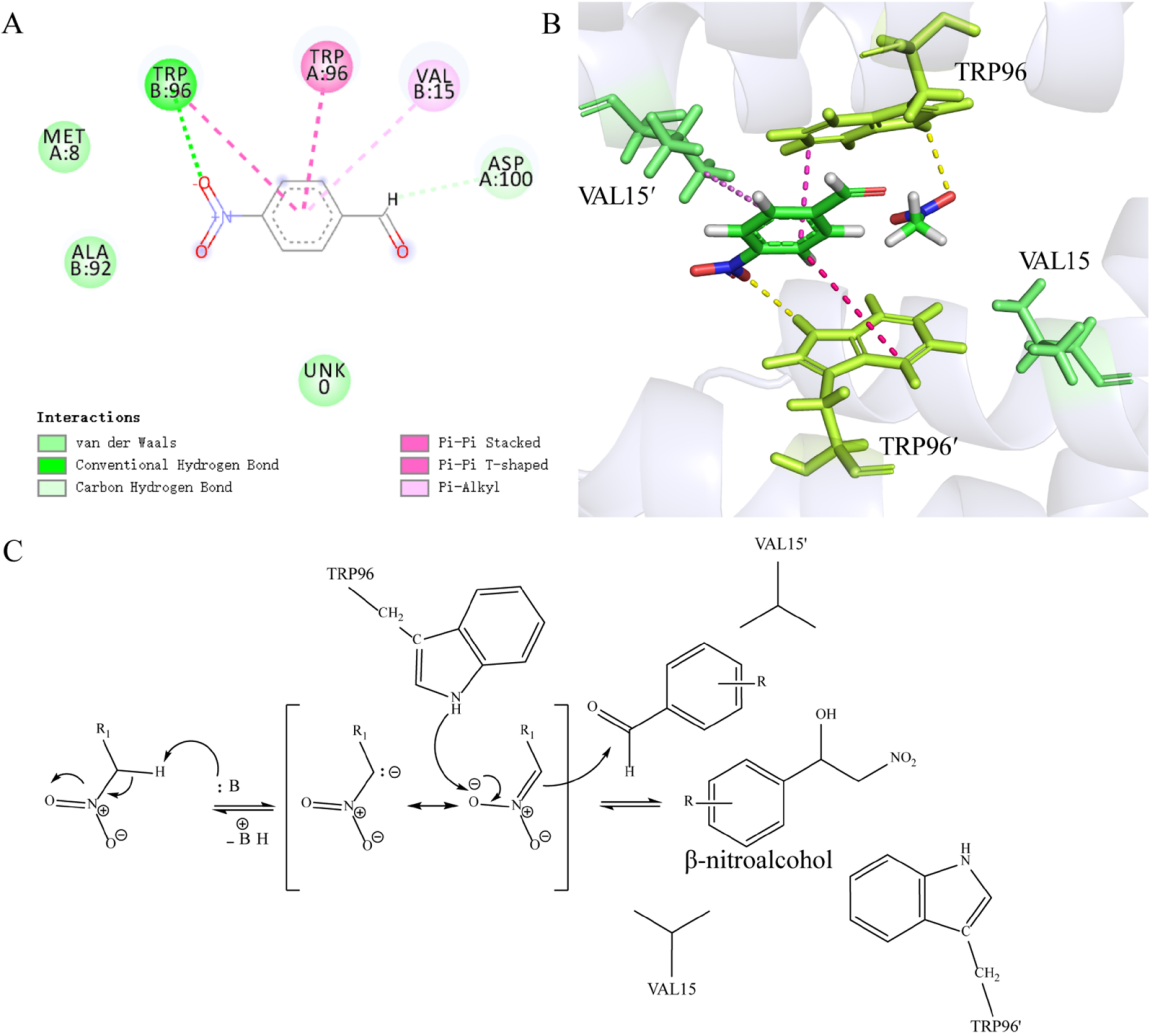
Analysis of enzyme catalysis mechanism. **A** 2D diagram of binding pocket; **B** 3D diagram of binding pocket; **C** Possible mechanism of LmrR-catalyzed Henry reaction. TRP 96ʹ corresponds to TRP B:96 in image A; VAL 15 corresponds to VAL A:15 in image A

Based on the spatial structure of LmrR, molecular docking results, and the screening results of the variant library, we hypothesized the mechanism of LmrR catalyzing Henry reaction. Two substrates (p-nitrobenzaldehyde and nitromethane) were enveloped in the space formed by four residues (V15, W96, V15ʹ, and W96ʹ) of LmrR. Surrounding the p-nitrobenzaldehyde were many interacting amino acids (D100, V15ʹ, W96, W96ʹ, M8, and A92ʹ), among which W96ʹ formed a hydrogen bond with p-nitrobenzaldehyde, and W96, W96ʹ and V15ʹ fixed the substrate through Pi-Pi stacking and Pi-alkyl interactions (Fig. 4A). Nitromethane formed a hydrogen bond with W96 (Fig. 4B) and simultaneously utilized proteinʹs alkalinity to facilitate formation of a carbon anion (Trost et al. 2002). In the wild-type LmrR-catalyzed Henry reaction, although A11ʹ does not directly interact with the substrate, based on the results from screening mutant variants, mutations to arginine or histidine at this position effectively enhance the catalytic activity of LmrR. This enhancement suggests that positively charged groups, even when not directly involved in substrate binding, may contribute to the stabilization of the nitroalkane carbanion in the catalytic process (Laureanti et al. 2020). Steric hindrance from nearby amino acid residues constrained the attacking direction of the reaction, and when the distance between the carbon cation and carbon anion was less than 3.5 Å (Liu et al. 2020), Nitromethane attacked p-nitrobenzaldehyde, which interacted with the surrounding amino acids, and then Henry reaction occurred (Fig. 4C) (Ching and Kallen 1978).

### Site-specific incorporation of non-canonical amino acids

**Fig. 5:**
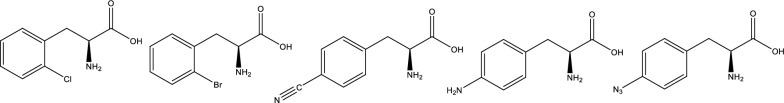
Five non-canonical amino acids

The molecular docking results showed that two substrates were contained within the space formed by the two sites Val15 and Trp96 on the two subunits of LmrR, and that the variant V15F improved enzyme activity. Therefore, this experiment further investigated whether larger and diverse electrostatic-potential-rich non-canonical amino acids (phenylalanine derivatives) could enhance LmrR catalytic efficiency (Green et al. 2016). The five phenylalanine derivatives included halogenated 2-chloro-L-phenylalanine and 2-bromo-L-phenylalanine (Br), as well as polar 4-cyan-L-phenylalanine (CNF), 4-amino-L-phenylalanine (pAF), and 4-azido-L-phenylalanine (AzF) (Fig. 5).

**Fig. 6:**
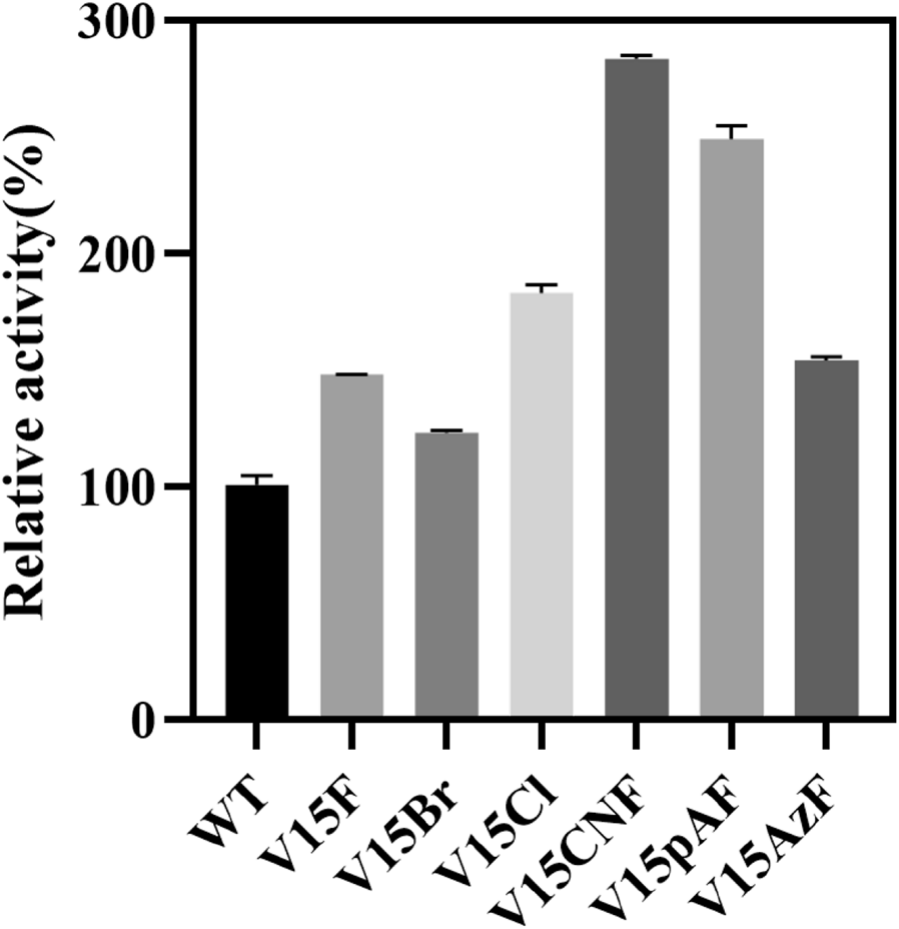
Comparison of enzyme activity between wild type and variants of LmrR

This experiment used a uniform concentration of pure enzyme to catalyze Henry reaction. The relative enzyme activity of other variants was calculated based on the wild-type enzyme activity set at 100%, as shown in Fig. 6. The study found that the enzyme activity of the V15F variant was 1.48 times that of the wild-type. Compared to the wild-type, introducing non-canonical amino acids at position 15 in LmrR could improve enzyme activity. The order of efficiency in improving enzyme activity from high to low was CNF, pAF, Cl, AzF, and Br, with their enzyme activities being 2.84, 2.49, 1.83, 1.54, and 1.23 times that of the wild-type, respectively. Except for V15Br, the variants introduced with non-canonical amino acids had better catalytic effects than V15F, indicating that site-specific introduction of non-canonical amino acids can enhance enzyme activity, and the effect of introducing non-canonical amino acids was better than that of conventional natural amino acid mutations. Compared to mutations involving natural amino acids, the introduction of phenylalanine analogs with active groups, such as cyano and amino groups, stabilizes the nitroalkane near the active site due to these positively charged groups, increasing the collision frequency of the two substrates. This indirectly confirms that mutating alanine at position 11 to an amino acid with a positively charged side chain similarly enhances the enzymatic activity of LmrR.

### Enzyme property of LmrR and its variants

**Fig. 7:**
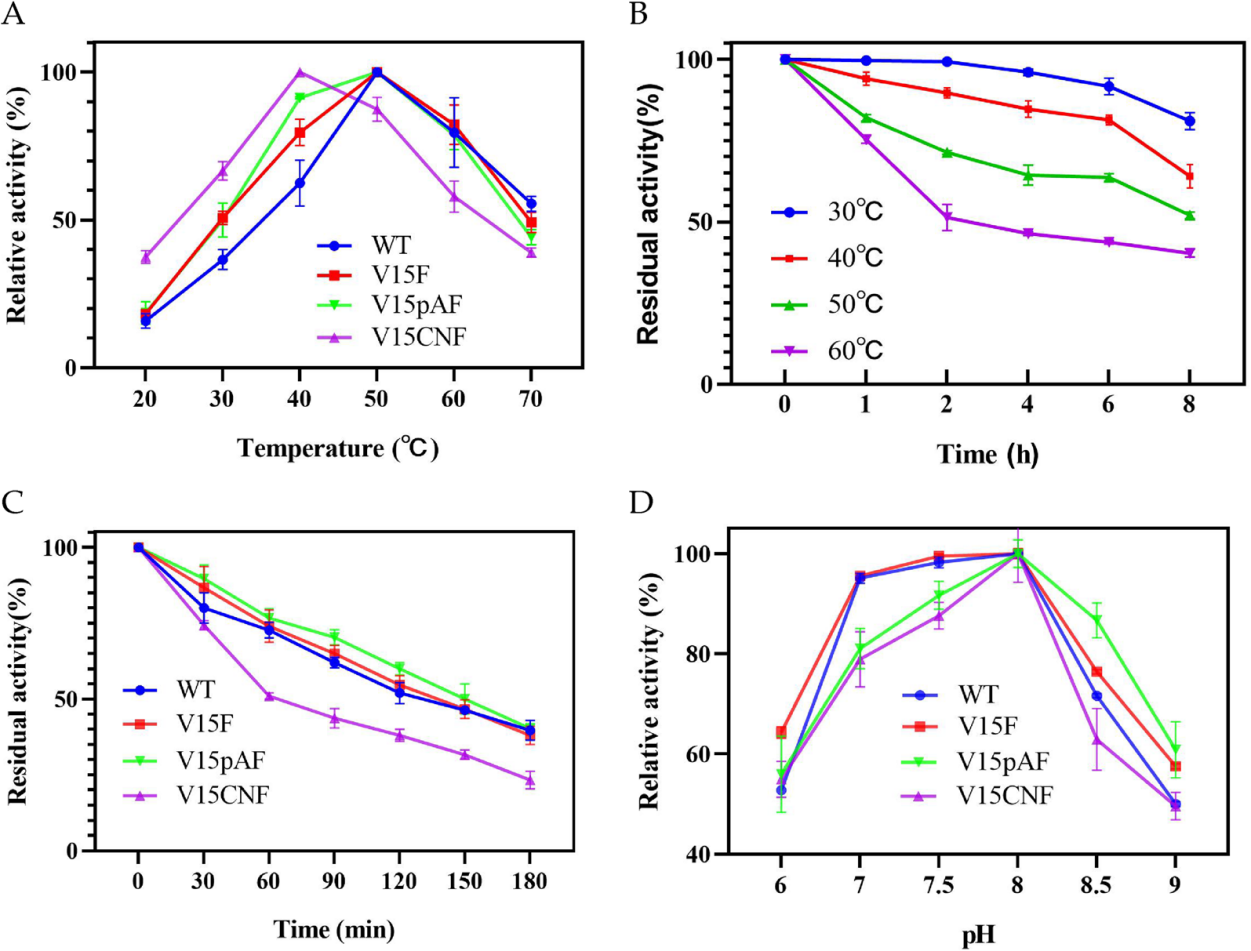
Characterization of wild-type and variants of LmrR. **A** Effects of temperature on the catalytic activity. Reaction condition: 1 mL of water containing purified wild-type and variants (0.3 mg/mL), p-nitrobenzaldehyde (12 mM), nitromethane (48 mM) at 20 °C, 30 °C, 40 °C, 50 °C, 60 °C, and 70 °C; **B** Thermal stability of LmrR at different temperatures. The WT incubated in the water at 30 °C, 40 °C, 50 °C, 60 °C, respectively. Reaction conditions: 1 mL of water containing purified WT (0.3 mg/mL), p-nitrobenzaldehyde (12 mM), nitromethane (48 mM) at 40 °C; **C** Comparison of thermal stability of wild type and variants of LmrR. he WT and different variants were incubated in the water at 60 ℃ for 30 min, 60 min, 90 min, 120 min, 150 min, and 180 min, respectively. Reaction condition: 1 mL of water containing purified wild-type and variants (0.3 mg/mL), p-nitrobenzaldehyde (12 mM), nitromethane (48 mM) at 40 °C; **D** Effects of pH on the catalytic activity. Reaction condition: 1 mL of different buffer (pH 6.0, 7.0, 7.5, 8.0, 8.5, 9.0) containing purified wild-type and variants (0.3 mg/mL), p-nitrobenzaldehyde (12 mM), nitromethane (48 mM) at 40 °C

The effect of temperature on the catalytic reaction of variants was shown in Fig. 7A, where optimal temperatures for LmrR wild type (WT) and variants V15F and V15pAF were around 50 °C, while optimal temperature for variant V15CNF was around 40 °C. It was speculated that the reason for this phenomenon was that V15CNF increased the enzyme's catalytic activity but decreased its structural stability.

Temperature affects thermal stability of enzyme catalysis by altering the spatial structure of proteins. Enzymes are prone to denaturation and inactivation under high temperature conditions. With an initial enzyme activity of 100%, the thermal stability of WT was calculated at 30 °C, 40 °C, 50 °C, and 60 °C. As shown in Fig. 7B, WT exhibited good thermal stability below 50 °C. After incubation in a water bath at 30 °C for 4 h, the enzyme activity of WT did not significantly decrease, and after 6 h, the remaining enzyme activity was higher than 90%. After incubation at 40 °C for 6 h, the residual enzyme activity was higher than 80%. However, when the temperature exceeded 50 °C, the decrease in enzyme activity gradually increased. After incubation at 60 °C for 3 h, the remaining enzyme activity was less than 50%.

The thermal stability of different variants was tested at 60 °C, and the results are shown in Fig. 7C. The decline in enzyme activity of WT, V15F, and V15pAF variants was relatively gentle, while that of V15CNF variant was severe. After incubation at 60 °C for 90 min, the residual enzyme activity of V15pAF was 70%, V15F was 65%, WT was 62%, and V15CNF was 44%. The significant decline in thermal stability of V15CNF variant suggested that there was a trade-off between enzyme catalytic activity and stability in V15CNF mutation.

The effect of pH on the catalytic activity of different variant enzymes was shown in Fig. 7D. The enzyme activity of variants followed the same trend as the wild-type, with an initial increase and then a decrease as pH increased, and their optimal pH was 8. Compared to the wild-type, the V15CNF curve narrowed, indicating that changes of pH had a more significant impact on the structure of the variant V15CNF.

The kinetic parameters of wild-type and variants of LmrR catalyzing Henry reaction were shown in Table 4. It could be seen that the *k*_cat_ values of V15CNF, V15pAF, and V15F were 1.87, 1.67, and 1.52 times that of the wild-type, respectively. The *K*_m_ values of V15pAF and V15F were lower than that of the wild-type, indicating that these two mutations increased the enzyme's affinity for the substrate (p-nitrobenzaldehyde). On the other hand, the *K*_m_ of V15CNF was increased, indicating a decreased affinity for the substrate (p-nitrobenzaldehyde), which resulted in smaller increase in *k*_cat_/*K*_m_.

**Table 4:** Kinetic parameters of wild type and variants of LmrR for Henry reaction

Mutation	V_max_ (mM/min)	*K*_m_ (mM)	*k*_cat_ (min^−1^)	*k*_cat_/ *K*_m_ (mM^−1^ min^−1^)
LmrR	1.29	9.30	58.05	6.24
V15F	1.96	8.28	88.20	10.65
V15CNF	2.41	10.08	108.45	10.76
V15pAF	2.15	8.86	96.75	10.92

### Spectrum of Henry reaction

The substrate spectrum of LmrR-V15CNF-catalyzed Henry reaction was investigated, and the results were shown in Table 5. LmrR-V15CNF could catalyze the reaction between aromatic aldehydes with different substituents on the phenyl ring and nitromethane, with product yielded ranging from 55 to 99%. Furthermore, benzaldehydes substituted with strong electron-withdrawing groups (such as nitrobenzaldehyde) were more reactive, and the para (p-) position was more reactive than the ortho (o-) or meta (m-) positions, possibly due to steric hindrance. The para position had less steric hindrance, which made nitromethane more prone to nucleophilic attack. Additionally, it was more compatible with the enzyme's spatial structure and was favorable for interaction with the enzyme. The product yield of the bromine group with weak electron-withdrawing properties was lower than that of the strong electron-withdrawing nitro group, but higher than that of the unsubstituted (-H) and electron-donating methyl groups. This might be due to the fact that the electron-donating group reduced the electrophilicity of the carbonyl carbon atom, leading to a decrease in its reactivity.

**Table 5:** Reactant scope of V15CNF for Henry reaction

			
Entry	R	Time^a^ /h	Yield^b^ /%
1	p-NO_2_	8	99.2
2	m-NO_2_	8	97.8
3	o-NO_2_	8	98.4
4	p-Br	8	66.7
5	p-CH_3_	8	57.4
6	H	8	55.6

^a^Reaction conditions: aldehyde (12 mmol/L), nitromethane (48 mmol/L), V15CNF (0.3 mg) and buffer (1 mL, pH 8.0) at 40 °C for 8 h

^b^Yield of product detected by HPLC

### Molecular dynamics analysis of mechanism for improving enzyme activity

**Fig. 8:**
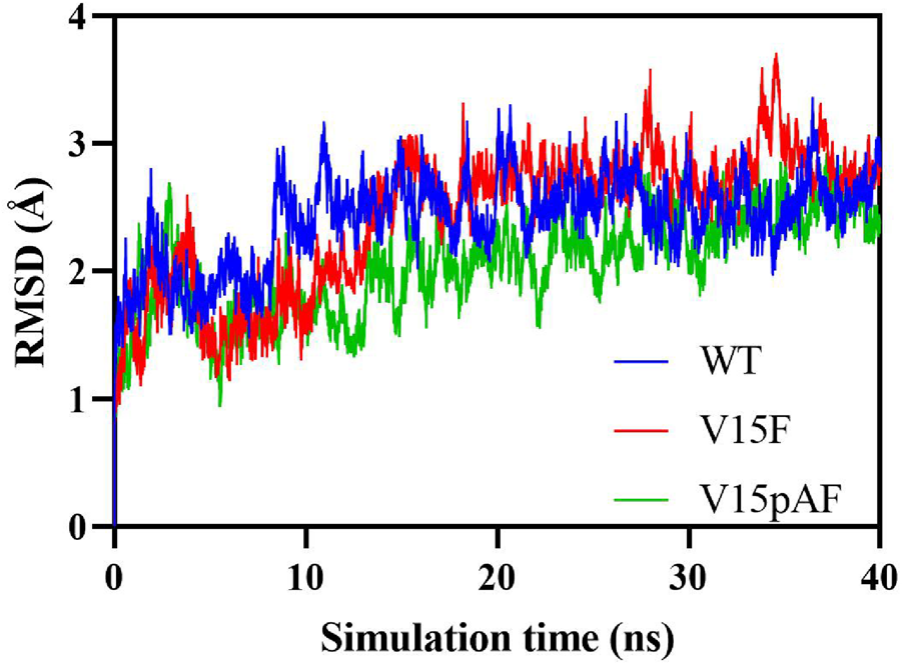
Temporal variation of RMSD values in wild type and variants of LmrR

**Fig. 9:**
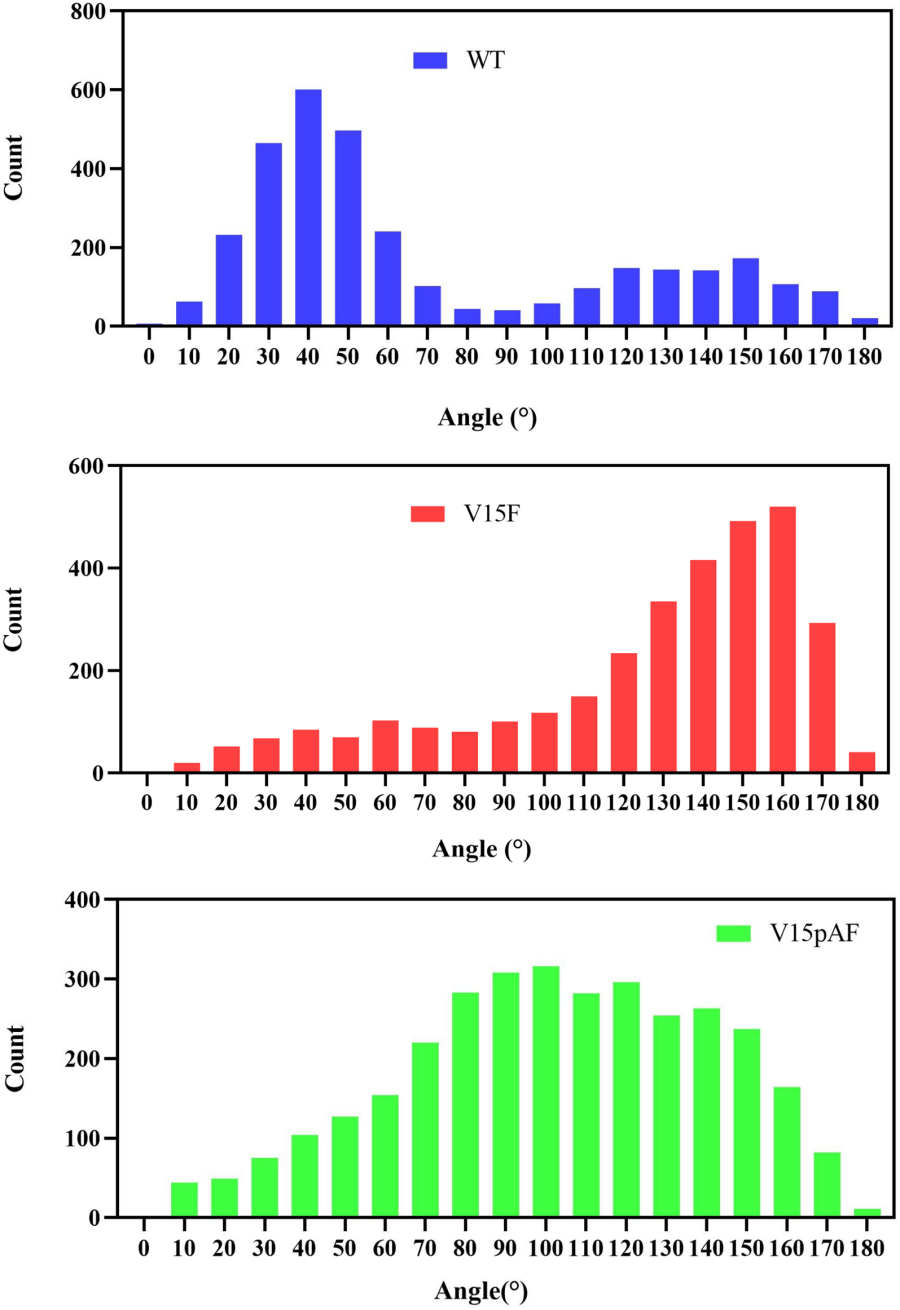
Statistical analysis of nucleophilic attack Angle in wild type and variants

In this study, molecular dynamics simulations were performed for WT, V15F, and V15pAF. The RMSD data showed that the simulation time of 40 ns was sufficient for systems to become stable (Fig. 8). The attacking angles in the 3000 conformations of each of the three dynamics models were statistically analyzed, as shown in Fig. 9. Studies have shown that the optimal angle range for nucleophilic attack on sp^2^-hybridized electrophilic centers is 100°–110° (Liu et al. 2020). The Burgi-Dunitz orbital angle is used to determine the nucleophilic attack angle of nucleophilic reagents on carbonyl carbons, which results from the overlap of the lowest unoccupied molecular orbital (LUMO) of the carbonyl and the highest occupied molecular orbital (HOMO) of the nucleophilic reagent (Dunitz et al. 1974). The proportion of conformations of V15pAF matching the Burgi-Dunitz angle was 18.3%, which was 3.9 times that of WT, and the proportion of conformations of V15F matching the Burgi-Dunitz angle was 8.2%, which was 1.7 times that of WT. This demonstrated that V15pAF and V15F had more effective nucleophilic attack angles that correspond to the orientation of the carbonyl π orbital, and the improvement in catalytic activity of the variants might be attributed to an increase in the interaction between HOMO and LUMO.

The proportion of amino acids that interacted with two substrates and had attack distances smaller than 3.5 Å was calculated (Table 6). The number of amino acids that interacted with two substrates in WT was 9, while in V15F and V15pAF it was 12, indicating that V15F and V15pAF strengthened the amino acid network around the substrates. The proportion of attack distances smaller than 3.5 Å in V15F and V15pAF increased by 1 and 1.62 times compared to WT, respectively, indicating that Henry reaction was more likely to occur in the variant catalytic system. Overall, it was found that V15F and V15pAF enhanced the amino acid network around the substrates, bringing the two substrates closer together and increasing collision frequency, thereby improving enzyme activity. Molecular dynamics simulation results confirmed the assumption that interaction between the enzyme and substrates was related to Henry reaction.

**Table 6:** Statistical analysis of surrounding amino acids and C–C distances

Mutation	Number of amino acids around nitromethane	Number of amino acids around p-nitrobenzaldehyde	The proportion of C–C distance < 3.5 Å (%)
WT	4	6	1.62
V15F	5	7	3.25
V15pAF	5	7	4.25

## Conclusion

The key amino acid residues W96 and V15 that catalyze Henry reaction of LmrR were determined through alanine scanning, and five enzyme activity-enhancing mutations were successfully obtained through saturation mutagenesis scanning: A11K, A11H, A11M, V15F, and A92R, which increased enzyme activity by 30%, 25%, 20%, 36%, and 22%, respectively. In cases where the mechanism of enzyme-catalyzed Henry reaction is unclear, rational enzyme design assisted by computer is employed. This strategy aims to enhance the affinity between the enzyme and substrate, thereby improving enzyme catalytic activity. It provides an approach for enzyme activity modification and the design of new enzymes in situations where the mechanism is not well understood. Five non-canonical amino acids were site-specifically introduced into LmrR to further improve catalytic activity. The enzyme activities of these five variants (V15CNF, V15pAF, V15Cl, V15AzF, and V15Br), which contain non-canonical amino acids, were 184%, 149%, 83%, 54%, and 23% higher than that of the wild type, respectively. Introducing non-canonical amino acids with immense structural and electrostatic diversity breaks through the limitations of natural amino acid residues, providing proteins with improved catalytic properties.

In summary, by utilizing enzyme-catalyzed Henry reaction, we primarily utilize computer-assisted genetic optimization rather than traditional chemical optimization based on reaction conditions to modify enzyme activity. The results indicate that genetic optimization not only enhances enzyme activity but also provides insights into the reaction mechanism. While rational engineering of LmrR and the incorporation of non-natural amino acids have successfully enhanced enzymatic activity, it is regrettable that selective changes were not identified during these processes. Subsequent research will continue to employ strategies involving the modification of non-natural amino acids, introducing non-natural amino acids with larger spatial hindrance, or incorporating multiple non-natural amino acids to achieve single-enzyme asymmetric catalysis in the Henry reaction.

## Materials and methods

### Design of variants

The amino acid sequence of LmrR was imported into Discovery Studio software to find the appropriate mutation sites. LmrR crystal structure was downloaded from the PDB database (PDB code: 3F8F). Original ligands and water molecules from 3F8F were removed. CDOCKER model was used to dock substrates into the active pocket of LmrR. After choosing the perfect binding position, a sphere was drawn around. For all the amino acids located inside the sphere, alanine scanning was applied to identify their functional contributions; the results were shown in Additional file 1: Table S1. After alanine scanning mutagenesis, residues Trp96, Trp96′, Val15, Ala92′, Ala11′, Val15′, Asp100, Met8 and Met8′ were randomly replaced by saturation mutation to further ensure function of these sites, and the results were listed in Additional file 1: Table S2.

### Site-directed mutagenesis of LmrR

A Stratagene QuikChange method was used to carry out site-directed mutagenesis with the LmrR gene in the plasmid pET-17b as the template (Georgescu et al. 2003). The primers used were listed in Additional file 1: Table S3. 25 μL of PrimeSTAR Max premix (2 ×), 1 μL forward primer, 1 μL reverse primer, 0.5 μL LmrR DNA, and 22.5 μL pure water. The PCR program was as follows: 98 °C for 2.5 min, followed by 30 cycles of 98 °C for 15 s, 60 °C for 15 s, 72 °C for 1.5 min, and a final extension at 72 °C for 5 min. The final products were digested with DpnI at 37 °C for 1 h to remove the parental plasmid. Purified products were then transformed into competent E. coli BL21 (DE3) cells by heat shock at 42 °C for 90 s in a water bath.

### Protein expression and purification

BL21 (DE3) harboring the pET-17b-LmrR was incubated in LB medium containing Ampicillin (100 μg/mL) at 37 °C and 200 rpm. When the OD600 reached 0.6−0.8, isopropyl β-D-1-thiogalactopyranoside (IPTG) was added at a final concentration of 0.5 mM, and protein expression was induced at 18 °C for 18 h. The cells were harvested by centrifugation at 4000 rpm for 20 min, resuspended in pure water. After sonication, the cell extract was centrifuged at 10,000 rpm at 4 °C for 10 min, and the supernatant was collected. The recombinant LmrR was purified using Ni–NTA resin, and SDS-PAGE used to analyze the purity of the protein preparations. Protein concentrations were measured by Nanodrop.

### Detection of products and determination of enzyme activity

#### Detection of β-nitroalcohol

The products were detected using high-performance liquid chromatography (HPLC). The HPLC equipment was FL 2200. The chromatographic column used was a CHIRALPAK IB N-5, which was a 4.6 mm × 250 mm chiral chromatographic column. The mobile phase ratio was n-hexane: isopropanol = 80:20. The detection wavelength was set at 260 nm and the flow rate was 1 mL/min. The retention time for the substrate p-nitrobenzaldehyde was around 9 min, while the two stereoisomers of the product β-nitroalcohol eluted at 11 min and 13 min, respectively.

#### Generation of product standard curve

A series of β-nitroalcohol solutions of different concentrations (3 mM, 4.5 mM, 6 mM, 7.5 mM, 9 mM, 12 mM) were prepared. The solutions were filtered through 0.25 μm organic filter membranes, and the peak area was measured using HPLC. The β-nitroalcohol concentration was plotted on the X-axis, and the peak area was plotted on the Y-axis. A linear fit was performed, and the product β-nitroalcohol standard curve was obtained (y = 12.71x-0.2, R^2^ = 0.9994).

#### Catalysis system and evaluation indicators

Crude enzyme reaction: A total of 1 mL system containing 12 mM p-nitrobenzaldehyde, 48 mM nitromethane, crude enzyme and solvent water, was reacted at 30 °C in a metal bath for 10 min. 10 μL of the reaction solution was taken and diluted 50 times with isopropanol and filtered through 0.25 μm organic filter membranes. HPLC was used for analysis.

Pure enzyme reaction: A total of 1 mL system containing 12 mM p-nitrobenzaldehyde, 48 mM nitromethane, 0.3 mg/mL pure enzyme and solvent water, was reacted at 30 °C in a metal bath for 10 min. 10 μL of the reaction solution was taken and diluted 50 times with isopropanol and filtered through 0.25 μm organic filter membranes. HPLC was used for analysis, and the yield was calculated using formula:

$${\text{Yield}}\,{\text{(\% )}}\, = \,\left[ {{\text{product}}} \right]/\left[ {{\text{substrate}}} \right]{\text{int}}\, \times \,{1}00\%$$

### Incorporation of non-canonical amino acid, Purification, And Analysis

This experiment chose to introduce non-canonical amino acids through Stop codon suppression method (Yu et al. 2017). Plasmid pET17b-LmrR-V15TAG represented Val at position 15 was mutated to an amber codon for incorporating of non-canonical amino acids. Besides, Plasmid pET17b-LmrR-V15TAG contained 6XHis tag at C-terminus. Plasmid pET17b-LmrR-V15TAG and the plasmid containing the aminoacyl tRNA synthetase/tRNA pair that recognized the amber codon were co-transformed into E. coli BL21 (DE3) (Chatterjee et al. 2013; Schultz et al. 2006; Mehl et al. 2003; Chin et al. 2002) At 18 °C, added IPTG to induce protein expression for 20 h. If non-canonical amino acids were not present, LmrR was interrupted at position 15TAG, resulting in the inability to obtain full-length LmrR protein. However, when non-canonical amino acids were present, they underwent aminoacylation connection with an exogenous tRNA under the action of an exogenous aaRS. The exogenous tRNA bound to the UAG site, forming a complex of non-canonical amino acid-tRNA-mRNA. The corresponding non-canonical amino acid was then translated at the UAG site, resulting in the production of full-length LmrR protein. After purification using a C-terminal histidine tag and Ni-affinity chromatography, the protein was verified using SDS-PAGE gel electrophoresis.

### Analysis of enzyme properties

The optimum temperature was determined as follows. Equal amounts of purified wild-type and variants were taken, and the substrate concentration was set at 12 mM for p-nitrobenzaldehyde and 48 mM for nitromethane. Reactions were carried out at 20 °C, 30 °C, 40 °C, 50 °C, 60 °C, and 70 °C.

To determine the thermal stability of wild-type at different temperatures, equal amounts of purified WT were placed in water baths at temperatures of 30 °C, 40 °C, 50 °C, and 60 °C. At regular intervals, enzyme samples were taken for reaction, with the initial enzyme activity set at 100%.

To determine the thermal stability of wild-type and different variants at 60 °C, equal amounts of purified wild-type and variants were incubated at 60 °C. At regular intervals, enzyme samples were taken for reaction, with the initial enzyme activity set at 100%.

The optimum pH was determined as follows. Equal amounts of purified wild-type and variant were taken, and the substrate concentration was set at 12 mM for p-nitrobenzaldehyde and 48 mM for nitromethane. Experiments were conducted at a temperature of 40 °C in different buffer solutions at pH 6, 7, 7.5, 8, 8.5, and 9.

The kinetic parameters of wild-type enzyme and variants were determined by measuring the reaction rate with different concentrations of p-nitrobenzaldehyde from 3 to 18 mM used for the reaction, while the concentration of nitromethane was set as excess at 1 M. The purified enzyme was added at a final concentration of 0.3 mg/mL and reactions were carried out at 40 °C and 750 rpm for 5 min. The reaction rates of LmrR at different substrate p-nitrobenzaldehyde concentrations were determined. The kinetics parameters were determined using the Michaelis−Menten equation.

### Synthesis and detection of standard samples of β-nitroalcohols

A solution of aldehyde (10 mmol) and nitromethane (1.1 mL, 20 mmol) in hexane was prepared, followed by addition of triethylamine (70 µL, 0.50 mmol). The reaction mixture was stirred at room temperature, and progress was monitored by thin-layer chromatography (TLC). After completion of the reaction, the mixture was neutralized with hydrochloric acid and washed with water and brine. Anhydrous magnesium sulfate was added for drying, followed by vacuum concentration. The purified product was obtained by silica gel column chromatography using a mixture of hexane and ethyl acetate (20:1 to 4:1) as the eluent. The corresponding β-nitroalcohol was obtained. In order to confirm synthesized products, the purified substances were subjected to hydrogen nuclear magnetic resonance (^1^H NMR) analysis and spectrums can be found in Additional file 1: Fig. S1. The HPLC conditions of β-nitroalcohols were shown in Additional file 1: Table S4. The liquid chromatography spectrums of reactions were shown in Additional file 1: Fig. S2.

### Saturation mutagenesis scan

Perform saturation mutagenesis scans using Calculate Mutation Energy Binding in Discovery Studio 4.0. After alanine scanning, saturate mutate the key residues to further ensure their functionality. Specific steps involve importing the docking results of the protein and ligand into Discovery Studio 4.0, applying the CHARMM force field to the protein using the "Apply Forcefield" program, selecting key residues for mutation to the remaining 19 amino acid residues, and calculating the difference in substrate affinity between the mutant and wild-type. The specific formula is as follows:

$$\begin{array}{c}\Delta \Delta {{\text{G}}}_{mut}=\Delta \Delta G\left({\text{mutant}}\right)-\Delta \Delta G\left(\mathrm{wild type}\right)\end{array}$$

### Molecular dynamics calculations

The initial conformations obtained from docking LmrR, LmrR-V15F variant, and LmrR-V15pAF (PDB code: 6I8N) with two ligands (p-nitrobenzaldehyde and nitromethane) through the CDOCKER module of Discovery Studio 4.0 were subjected to molecular dynamics simulations using the Sander program from Amber Tools 22 for 40 ns. The system employed a TIP3P water model and was neutralized with Na^+^ to make the system electrically neutral. The protein was parameterized using the ff14SB force field, while the ligands were parameterized using the general Amber force field (GAFF). The simulations started with energy minimization using steepest descent and conjugate gradient algorithms, followed by heating to 300 K under constant temperature and pressure conditions, equilibrating for 20 ns, and sampling for 40 ns. The particle mesh Ewald (PME) method with a cutoff of 10 Å was used to handle long-range electrostatic interactions and non-bonded interactions. Amber Tools 22 and VMD-1.9.3 were used for analysis and calculation of the dynamic trajectories, and graphical representations were generated using Pymol 2.5.

### Supplementary Information

**Additional file 1: Table S1.** Alanine scanning of LmrR. **Table S2.** Saturation scanning of LmrR. **Table S3.** Primer sequences of different mutation sites. **Table S4.** HPLC detection conditions of β-nitroalcohols. **Fig. S1.** 1H NMR spectrums of β-nitroalcohols. **Fig. S2.** Liquid chromatography spectrums of β-nitroalcohols.

## Data Availability

All data generated or analyzed during this study are included in this published article (and its supplementary information files).
