# Supplementary material for: Rationally introducing non-canonical amino acids to enhance catalytic activity of LmrR for Henry reaction
Source: Bioresour Bioprocess. 2024 Feb 29;11(1):26. doi: 10.1186/s40643-024-00744-w (PMC10992053; doi:10.1186/s40643-024-00744-w)
Supplement: Supplementary file 1 — Additional file 1: Table S1. Alanine scanning of LmrR. Table S2. Saturation scanning of LmrR. Table S3. Primer sequences of different mutation sites. Table S4. HPLC detection conditions of β-nitroalcohols. Fig. S1. 1H NMR spectrums of β-nitroalcohols. Fig. S2. Liquid chromatography spectrums of β-nitroalcohols. [file 40643_2024_744_MOESM1_ESM.docx]

**Additional Information**

Rationally introducing non-canonical amino acids to enhance catalytic activity of LmrR for Henry reaction

*Lan Wang^a#^, Mengting Zhang^a#^, Haidong Teng^a^, Zhe Wang^b^, Shulin Wang^a^, Pengcheng Li^a^, Jianping Wu^a^, Lirong Yang^a^ and Gang Xu^a^**

^a^ Institute of Bioengineering, College of Chemical and Biological Engineering, Zhejiang University, Hangzhou, Zhejiang 310027, China.

^b^ Huadong Medicine Co., Ltd, Hangzhou, Zhejiang 310011, China.

^#^ These authors contributed equally to the article.

*Correspondence and requests for materials should be addressed to Gang Xu (Email: xugang_1030@zju.edu.cn)

**Contents**

[**Additional Tables** **2**](#_Toc154428807)

[Table S1 Alanine scanning of LmrR 2](#_Toc154428808)

[Table S2 Saturation scanning of LmrR 5](#_Toc154428809)

[Table S3 Primer sequences of different mutation sites 10](#_Toc154428810)

[Table S4 HPLC detection conditions of β-nitroalcohols 12](#_Toc154428811)

[**Additional Figures** **13**](#_Toc154428812)

[Fig. S1 ^1^H NMR spectrums of β-nitroalcohols 14](#_Toc154428813)

[Fig. S2 Liquid chromatography spectrums of β-nitroalcohols 15](#_Toc154428814)

# Additional Tables

## Table S1 Alanine scanning of LmrR

| Mutation | Mutation Energy (kcal/mol) | Effect |
| --- | --- | --- |
| A:TRP96>ALA | 2.43 | DESTABILIZING |
| B:TRP96>ALA | 1.51 | DESTABILIZING |
| B:VAL15>ALA | 0.67 | DESTABILIZING |
| A:ASP100>ALA | 0.41 | NEUTRAL |
| A:MET8>ALA | 0.37 | NEUTRAL |
| B:MET8>ALA | 0.23 | NEUTRAL |
| A:ILE103>ALA | 0.14 | NEUTRAL |
| A:VAL15>ALA | 0.09 | NEUTRAL |
| A:VAL99>ALA | 0.09 | NEUTRAL |
| A:GLN12>ALA | 0.05 | NEUTRAL |
| B:PRO5>ALA | 0.05 | NEUTRAL |
| B:GLN12>ALA | 0.05 | NEUTRAL |
| B:ASN19>ALA | 0.05 | NEUTRAL |
| B:LEU18>ALA | 0.04 | NEUTRAL |
| B:PHE93>ALA | 0.04 | NEUTRAL |
| A:ILE4>ALA | 0.03 | NEUTRAL |
| B:ILE16>ALA | 0.03 | NEUTRAL |
| B:MET89>ALA | 0.03 | NEUTRAL |
| A:PHE93>ALA | 0.02 | NEUTRAL |
| A:GLU94>ALA | 0.02 | NEUTRAL |
| B:ILE103>ALA | 0.02 | NEUTRAL |
| A:PRO5>ALA | 0.01 | NEUTRAL |
| A:ASN19>ALA | 0.01 | NEUTRAL |
| A:MET89>ALA | 0.01 | NEUTRAL |
| A:SER97>ALA | 0.01 | NEUTRAL |
| A:ARG98>ALA | 0.01 | NEUTRAL |
| A:ILE102>ALA | 0.01 | NEUTRAL |
| A:GLU104>ALA | 0.01 | NEUTRAL |
| A:GLU107>ALA | 0.01 | NEUTRAL |
| B:LYS6>ALA | 0.01 | NEUTRAL |
| B:ASN14>ALA | 0.01 | NEUTRAL |
| B:LEU57>ALA | 0.01 | NEUTRAL |
| B:ASN88>ALA | 0.01 | NEUTRAL |
| B:LEU91>ALA | 0.01 | NEUTRAL |
| B:GLU94>ALA | 0.01 | NEUTRAL |
| B:SER95>ALA | 0.01 | NEUTRAL |
| Mutation | Mutation Energy (kcal/mol) | Effect |
| B:VAL99>ALA | 0.01 | NEUTRAL |
| A:LEU18>ALA | 0 | NEUTRAL |
| A:ASN88>ALA | 0 | NEUTRAL |
| A:LEU91>ALA | 0 | NEUTRAL |
| A:SER95>ALA | 0 | NEUTRAL |
| A:LYS101>ALA | 0 | NEUTRAL |
| B:THR13>ALA | 0 | NEUTRAL |
| B:GLY85>ALA | 0 | NEUTRAL |
| B:HIS86>ALA | 0 | NEUTRAL |
| B:GLU87>ALA | 0 | NEUTRAL |
| B:ALA92>ALA | 0 | NEUTRAL |
| B:SER97>ALA | 0 | NEUTRAL |
| B:ARG98>ALA | 0 | NEUTRAL |
| A:ALA11>ALA | -0.01 | NEUTRAL |
| A:THR13>ALA | -0.01 | NEUTRAL |
| A:ALA92>ALA | -0.01 | NEUTRAL |
| B:ARG10>ALA | -0.01 | NEUTRAL |
| B:LEU17>ALA | -0.01 | NEUTRAL |
| B:LYS22>ALA | -0.01 | NEUTRAL |
| B:ARG90>ALA | -0.01 | NEUTRAL |
| B:ASP100>ALA | -0.03 | NEUTRAL |
| B:LEU9>ALA | -0.04 | NEUTRAL |
| A:ASN14>ALA | -0.06 | NEUTRAL |
| A:GLU7>ALA | -0.07 | NEUTRAL |
| A:ARG10>ALA | -0.07 | NEUTRAL |
| B:GLU7>ALA | -0.08 | NEUTRAL |
| B:ALA11>ALA | -0.08 | NEUTRAL |
| A:LEU9>ALA | -0.09 | NEUTRAL |
| A:ILE16>ALA | -0.11 | NEUTRAL |

## Table S2 Saturation scanning of LmrR

| Mutation | Mutation Energy (kcal/mol) | Effect |
| --- | --- | --- |
| A:MET8>ALA | 0.37 | NEUTRAL |
| A:MET8>ARG | -0.02 | NEUTRAL |
| A:MET8>ASN | 0.08 | NEUTRAL |
| A:MET8>ASP | 0.15 | NEUTRAL |
| A:MET8>CYS | 0.31 | NEUTRAL |
| A:MET8>GLN | -0.03 | NEUTRAL |
| A:MET8>GLU | 0.1 | NEUTRAL |
| A:MET8>GLY | 0.43 | NEUTRAL |
| A:MET8>HIS | 0.41 | NEUTRAL |
| A:MET8>ILE | 0.1 | NEUTRAL |
| A:MET8>LEU | -0.05 | NEUTRAL |
| A:MET8>LYS | -0.14 | NEUTRAL |
| A:MET8>MET | 0 | NEUTRAL |
| A:MET8>PHE | -0.01 | NEUTRAL |
| A:MET8>PRO | 0.27 | NEUTRAL |
| A:MET8>SER | 0.42 | NEUTRAL |
| A:MET8>THR | 0.29 | NEUTRAL |
| A:MET8>TRP | -0.03 | NEUTRAL |
| A:MET8>TYR | 0.05 | NEUTRAL |
| A:MET8>VAL | 0.25 | NEUTRAL |
| A:VAL15>ALA | 0.09 | NEUTRAL |
| A:VAL15>ARG | 0.39 | NEUTRAL |
| A:VAL15>ASN | 0.09 | NEUTRAL |
| A:VAL15>ASP | -0.09 | NEUTRAL |
| A:VAL15>CYS | 0.07 | NEUTRAL |
| A:VAL15>GLN | 0.34 | NEUTRAL |
| A:VAL15>GLU | 0.84 | DESTABILIZING |
| A:VAL15>GLY | 0.2 | NEUTRAL |
| A:VAL15>HIS | -0.34 | NEUTRAL |
| A:VAL15>ILE | -0.06 | NEUTRAL |
| A:VAL15>LEU | -0.29 | NEUTRAL |
| A:VAL15>LYS | -0.06 | NEUTRAL |
| A:VAL15>MET | 0.51 | DESTABILIZING |
| A:VAL15>PHE | -0.45 | NEUTRAL |
| A:VAL15>PRO | 0.06 | NEUTRAL |
| A:VAL15>SER | 0.18 | NEUTRAL |
| A:VAL15>THR | 0.08 | NEUTRAL |
| A:VAL15>TRP | -0.72 | STABILIZING |
| A:VAL15>TYR | -0.47 | NEUTRAL |
| Mutation | Mutation Energy (kcal/mol) | Effect |
| A:VAL15>VAL | -0.01 | NEUTRAL |
| A:TRP96>ALA | 2.43 | DESTABILIZING |
| A:TRP96>ARG | 2.54 | DESTABILIZING |
| A:TRP96>ASN | 1.98 | DESTABILIZING |
| A:TRP96>ASP | 2.07 | DESTABILIZING |
| A:TRP96>CYS | 2.34 | DESTABILIZING |
| A:TRP96>GLN | 2.48 | DESTABILIZING |
| A:TRP96>GLU | 2.75 | DESTABILIZING |
| A:TRP96>GLY | 2.67 | DESTABILIZING |
| A:TRP96>HIS | 1.5 | DESTABILIZING |
| A:TRP96>ILE | 2.36 | DESTABILIZING |
| A:TRP96>LEU | 1.84 | DESTABILIZING |
| A:TRP96>LYS | 1.56 | DESTABILIZING |
| A:TRP96>MET | 2.24 | DESTABILIZING |
| A:TRP96>PHE | 1.16 | DESTABILIZING |
| A:TRP96>PRO | 2.2 | DESTABILIZING |
| A:TRP96>SER | 2.55 | DESTABILIZING |
| A:TRP96>THR | 2.31 | DESTABILIZING |
| A:TRP96>TRP | 0 | NEUTRAL |
| A:TRP96>TYR | 0.92 | DESTABILIZING |
| A:TRP96>VAL | 2.19 | DESTABILIZING |
| A:ASP100>ALA | 0.41 | NEUTRAL |
| A:ASP100>ARG | 0.05 | NEUTRAL |
| A:ASP100>ASN | -0.01 | NEUTRAL |
| A:ASP100>ASP | 0.05 | NEUTRAL |
| A:ASP100>CYS | 0.24 | NEUTRAL |
| A:ASP100>GLN | 0.27 | NEUTRAL |
| A:ASP100>GLU | 0.2 | NEUTRAL |
| A:ASP100>GLY | 0.47 | NEUTRAL |
| A:ASP100>HIS | 0.1 | NEUTRAL |
| A:ASP100>ILE | 0.22 | NEUTRAL |
| A:ASP100>LEU | -0.01 | NEUTRAL |
| A:ASP100>LYS | 0.2 | NEUTRAL |
| A:ASP100>MET | 0.27 | NEUTRAL |
| A:ASP100>PHE | -0.21 | NEUTRAL |
| A:ASP100>PRO | 0.2 | NEUTRAL |
| A:ASP100>SER | 0.64 | DESTABILIZING |
| A:ASP100>THR | 0.52 | DESTABILIZING |
| A:ASP100>TRP | 0.28 | NEUTRAL |
| A:ASP100>TYR | -0.05 | NEUTRAL |
| A:ASP100>VAL | 0.26 | NEUTRAL |
| B:MET8>ALA | 0.23 | NEUTRAL |
| Mutation | Mutation Energy (kcal/mol) | Effect |
| B:MET8>ARG | 0.12 | NEUTRAL |
| B:MET8>ASN | -0.01 | NEUTRAL |
| B:MET8>ASP | 0.25 | NEUTRAL |
| B:MET8>CYS | 0.2 | NEUTRAL |
| B:MET8>GLN | -0.1 | NEUTRAL |
| B:MET8>GLU | 0.27 | NEUTRAL |
| B:MET8>GLY | 0.33 | NEUTRAL |
| B:MET8>HIS | 0.04 | NEUTRAL |
| B:MET8>ILE | 0.02 | NEUTRAL |
| B:MET8>LEU | -0.11 | NEUTRAL |
| B:MET8>LYS | 0 | NEUTRAL |
| B:MET8>MET | -0.01 | NEUTRAL |
| B:MET8>PHE | 0.33 | NEUTRAL |
| B:MET8>PRO | 0.1 | NEUTRAL |
| B:MET8>SER | 0.47 | NEUTRAL |
| B:MET8>THR | 0.35 | NEUTRAL |
| B:MET8>TRP | 0.46 | NEUTRAL |
| B:MET8>TYR | 0.7 | DESTABILIZING |
| B:MET8>VAL | 0.04 | NEUTRAL |
| B:ALA11>ALA | -0.08 | NEUTRAL |
| B:ALA11>ARG | -0.5 | NEUTRAL |
| B:ALA11>ASN | -0.48 | NEUTRAL |
| B:ALA11>ASP | -0.33 | NEUTRAL |
| B:ALA11>CYS | -0.08 | NEUTRAL |
| B:ALA11>GLN | -0.78 | STABILIZING |
| B:ALA11>GLU | -0.65 | STABILIZING |
| B:ALA11>GLY | 0.06 | NEUTRAL |
| B:ALA11>HIS | -0.82 | STABILIZING |
| B:ALA11>ILE | -0.19 | NEUTRAL |
| B:ALA11>LEU | -0.73 | STABILIZING |
| B:ALA11>LYS | -1.09 | STABILIZING |
| B:ALA11>MET | -0.81 | STABILIZING |
| B:ALA11>PHE | -0.92 | STABILIZING |
| B:ALA11>PRO | -0.18 | NEUTRAL |
| B:ALA11>SER | 0.01 | NEUTRAL |
| B:ALA11>THR | -0.14 | NEUTRAL |
| B:ALA11>TRP | -0.41 | NEUTRAL |
| B:ALA11>TYR | -0.33 | NEUTRAL |
| B:ALA11>VAL | -0.4 | NEUTRAL |
| B:VAL15>ALA | 0.67 | DESTABILIZING |
| B:VAL15>ARG | -0.13 | NEUTRAL |
| B:VAL15>ASN | -0.14 | NEUTRAL |
| Mutation | Mutation Energy (kcal/mol) | Effect |
| B:VAL15>ASP | 0.35 | NEUTRAL |
| B:VAL15>CYS | 0.45 | NEUTRAL |
| B:VAL15>GLN | 0.58 | DESTABILIZING |
| B:VAL15>GLU | 1.06 | DESTABILIZING |
| B:VAL15>GLY | 0.96 | DESTABILIZING |
| B:VAL15>HIS | -0.08 | NEUTRAL |
| B:VAL15>ILE | 0.2 | NEUTRAL |
| B:VAL15>LEU | 0.21 | NEUTRAL |
| B:VAL15>LYS | 0.44 | NEUTRAL |
| B:VAL15>MET | 0.5 | NEUTRAL |
| B:VAL15>PHE | -0.45 | NEUTRAL |
| B:VAL15>PRO | 0.17 | NEUTRAL |
| B:VAL15>SER | 0.56 | DESTABILIZING |
| B:VAL15>THR | 0.37 | NEUTRAL |
| B:VAL15>TRP | -0.82 | STABILIZING |
| B:VAL15>TYR | -0.44 | NEUTRAL |
| B:VAL15>VAL | 0 | NEUTRAL |
| B:ALA92>ALA | 0 | NEUTRAL |
| B:ALA92>ARG | -0.52 | STABILIZING |
| B:ALA92>ASN | 0.01 | NEUTRAL |
| B:ALA92>ASP | -0.19 | NEUTRAL |
| B:ALA92>CYS | -0.01 | NEUTRAL |
| B:ALA92>GLN | -0.55 | STABILIZING |
| B:ALA92>GLU | -0.43 | NEUTRAL |
| B:ALA92>GLY | 0.26 | NEUTRAL |
| B:ALA92>HIS | -0.27 | NEUTRAL |
| B:ALA92>ILE | -0.12 | NEUTRAL |
| B:ALA92>LEU | -0.38 | NEUTRAL |
| B:ALA92>LYS | -0.41 | NEUTRAL |
| B:ALA92>MET | -0.22 | NEUTRAL |
| B:ALA92>PHE | -0.33 | NEUTRAL |
| B:ALA92>PRO | -0.28 | NEUTRAL |
| B:ALA92>SER | -0.04 | NEUTRAL |
| B:ALA92>THR | -0.22 | NEUTRAL |
| B:ALA92>TRP | -0.42 | NEUTRAL |
| B:ALA92>TYR | -0.4 | NEUTRAL |
| B:ALA92>VAL | -0.15 | NEUTRAL |
| B:TRP96>ALA | 1.51 | DESTABILIZING |
| B:TRP96>ARG | 0.66 | DESTABILIZING |
| B:TRP96>ASN | 1.2 | DESTABILIZING |
| B:TRP96>ASP | 1.23 | DESTABILIZING |
| B:TRP96>CYS | 1.36 | DESTABILIZING |
| Mutation | Mutation Energy (kcal/mol) | Effect |
| B:TRP96>GLN | 2.3 | DESTABILIZING |
| B:TRP96>GLU | 2.43 | DESTABILIZING |
| B:TRP96>GLY | 1.67 | DESTABILIZING |
| B:TRP96>HIS | 0.71 | DESTABILIZING |
| B:TRP96>ILE | 0.95 | DESTABILIZING |
| B:TRP96>LEU | 1.08 | DESTABILIZING |
| B:TRP96>LYS | 1.7 | DESTABILIZING |
| B:TRP96>MET | 1.63 | DESTABILIZING |
| B:TRP96>PHE | 0.45 | NEUTRAL |
| B:TRP96>PRO | 1.27 | DESTABILIZING |
| B:TRP96>SER | 1.87 | DESTABILIZING |
| B:TRP96>THR | 1.45 | DESTABILIZING |
| B:TRP96>TRP | -0.01 | NEUTRAL |
| B:TRP96>TYR | -0.04 | NEUTRAL |
| B:TRP96>VAL | 1.16 | DESTABILIZING |

## Table S3 Primer sequences of different mutation sites

| Mutation | Primer | 5' to 3' |
| --- | --- | --- |
| V15A | Forward Primer | GTGCTCAAACCAATgccATCCTGCTGAATG |
|  | Reverse Primer | ggcATTGGTTTGAGCACGCAGCATTTC |
| W96A | Forward Primer | TGGCGTTCGAATCCgcaAGTCGTGTGGAC |
|  | Reverse Primer | tgcGGATTCGAACGCCAGGCGCATGTTTTC |
| A11K | Forward Primer | GAAATGCTGCGTaaaCAAACCAATGTCATCC |
|  | Reverse Primer | tttACGCAGCATTTCTTTCGGGATTTC |
| A11F | Forward Primer | GAAATGCTGCGTgaaCAAACCAATGTCATC |
|  | Reverse Primer | ttcACGCAGCATTTCTTTCGGGATTTC |
| A11H | Forward Primer | GAAATGCTGCGTgtgCAAACCAATGTCATC |
|  | Reverse Primer | cacACGCAGCATTTCTTTCGGGATTTC |
| V15W | Forward Primer | GTGCTCAAACCAATTGGATCCTGCTGAATG |
|  | Reverse Primer | CCAATTGGTTTGAGCACGCAGCATTTC |
| V15F | Forward Primer | GTGCTCAAACCAATttcATCCTGCTGAATG |
|  | Reverse Primer | gaaATTGGTTTGAGCACGCAGCATTTC |
| A11Q | Forward Primer | GAAATGCTGCGTctgCAAACCAATGTCATCC |
|  | Reverse Primer | cagACGCAGCATTTCTTTCGGGATTTC |
| A11L | Forward Primer | GAAATGCTGCGTctgCAAACCAATGTCATCC |
|  | Reverse Primer | cagACGCAGCATTTCTTTCGGGATTTC |
| A11E | Forward Primer | GAAATGCTGCGTgaaCAAACCAATGTCATC |
|  | Reverse Primer | ttcACGCAGCATTTCTTTCGGGATTTC |
| A11W | Forward Primer | GAAATGCTGCGTtggCAAACCAATGTCATC |
|  | Reverse Primer | ccaACGCAGCATTTCTTTCGGGATTTC |
| A92W | Forward Primer | GAAAACATGCGCCTGtggTTCGAATCCTG |
|  | Reverse Primer | ccaCAGGCGCATGTTTTCATGGCCG |
| A92Q | Forward Primer | GAAAACATGCGCCTGctgTTCGAATCCTG |
|  | Reverse Primer | cagCAGGCGCATGTTTTCATGGCCG |
| A92R | Forward Primer | GAAAACATGCGCCTGagaTTCGAATCCTG |
|  | Reverse Primer | tctCAGGCGCATGTTTTCATGGCCG |
| R10A | Forward Primer | CCCGAAAGAAATGCTGgcgGCTCAAACCAATG |
|  | Reverse Primer | cgcCAGCATTTCTTTCGGGATTTCGGCACC |
| N14A | Forward Primer | GCGTGCTCAAACCgctGTCATCCTGCTG |
|  | Reverse Primer | agcGGTTTGAGCACGCAGCATTTCTTTC |
| Mutation | Primer | 5' to 3' |
| N19A | Forward Primer | GCGTGCTCAAACCgcgGTCATCCTGCTG |
|  | Reverse Primer | cgcGGTTTGAGCACGCAGCATTTCTTTC |
| M89A | Forward Primer | ATCGGCCATGAAAACgcaCGCCTGGCGTTC |
|  | Reverse Primer | tgcGTTTTCATGGCCGATTTCGGTCAGACG |
| F93A | Forward Primer | ATGCGCCTGGCGgcaGAATCCTGGAG |
|  | Reverse Primer | tgcCGCCAGGCGCATGTTTTCATGGC |
| D100A | Forward Primer | TCCTGGAGTCGTGTGgcaAAAATCATTG |
|  | Reverse Primer | tgcCACACGACTCCAGGATTCGAACGCCAG |
| V15TAG | Forward Primer | GTGCTCAAACCAATtagATCCTGCTGAATG |
|  | Reverse Primer | ctaATTGGTTTGAGCACGCAGCATTTC |

## Table S4 HPLC detection conditions of β-nitroalcohols

| β-nitroalcohol | column | | n-hexane/ isopropanol | flow rate | Wavelength | Column temperature | S -configuration retention time | | | R-configuration retention time |
| --- | --- | --- | --- | --- | --- | --- | --- | --- | --- | --- |
| \|  \| \| --- \| | | CHIRALPAK IB N-5 | 80/20 | 1 mL/min | 260 nm | 40°C | | 11.3 min | 13.2 min | |
| \|  \| \| --- \| | | CHIRALPAK IB N-5 | 80/20 | 1 mL/min | 260 nm | 40°C | | 10.7 min | 11.6 min | |
| \|  \| \| --- \| | | CHIRALPAK IB N-5 | 80/20 | 1 mL/min | 260 nm | 40°C | | 7.4 min | 7.8 min | |
| \|  \| \| --- \| | | CHIRALPAK IB N-5 | 80/20 | 1 mL/min | 260 nm | 40°C | | 8.3 min | 9.9 min | |
| \|  \| \| --- \| | | CHIRALPAK IB N-5 | 80/20 | 1 mL/min | 260 nm | 40°C | | 7.5 min | 8.7 min | |
| \|  \| \| --- \| | | CHIRALPAK IB N-5 | 65/35 | 1 mL/min | 260 nm | 40°C | | 6.3 min | 6.9 min | |

# Additional Figures

| A | B |
| --- | --- |
| **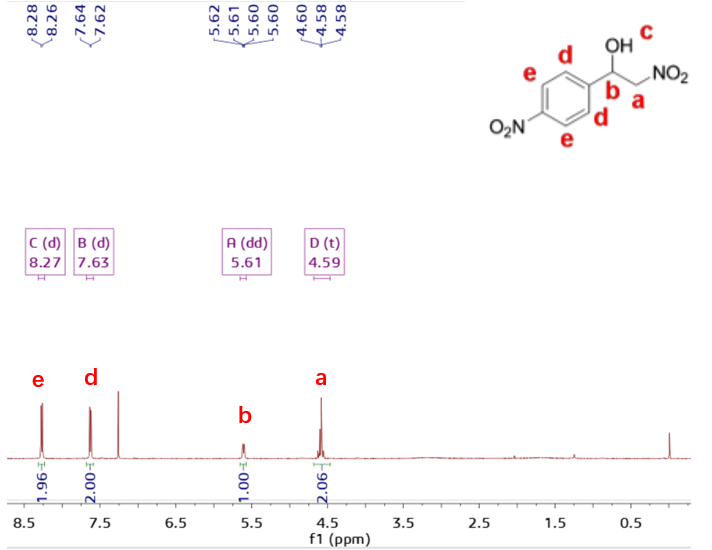** | 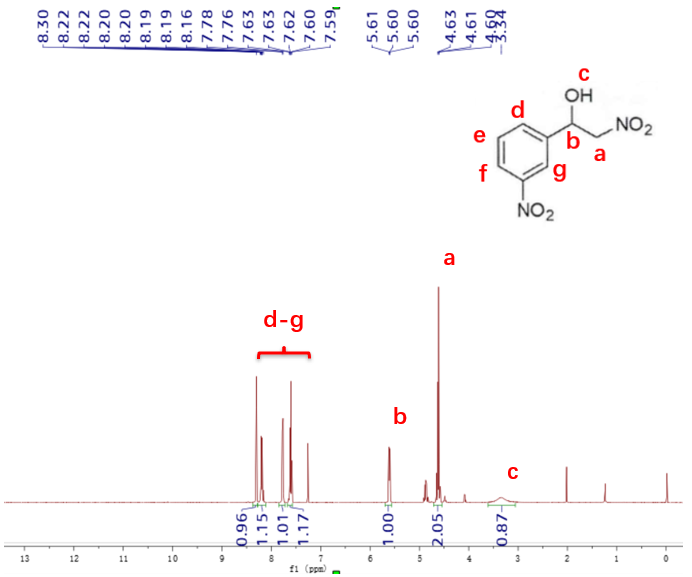 |
| C | D |
| **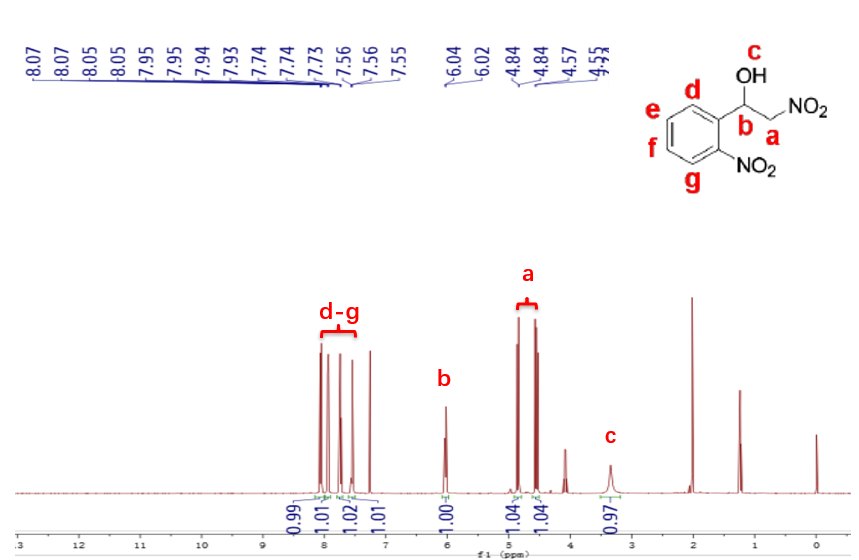** | **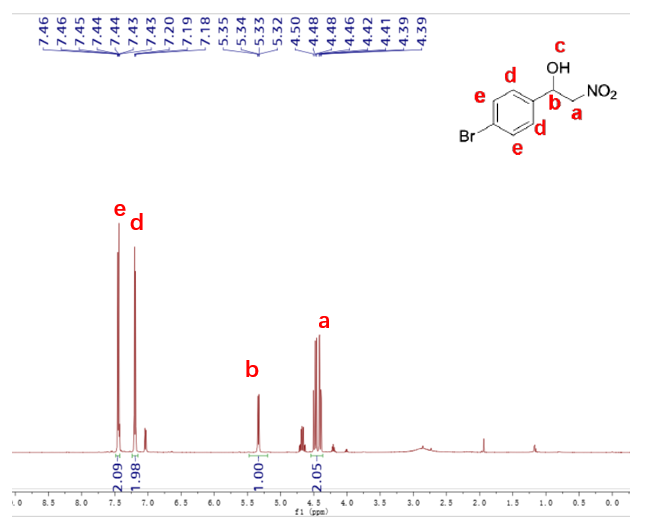** |
| E | F |
|  | **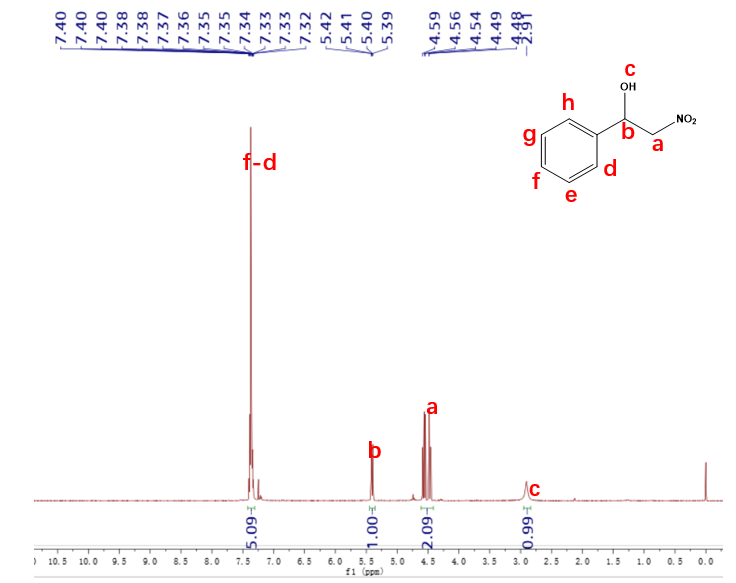** |

## Fig. S1 ^1^H NMR spectrums of β-nitroalcohols. (A) 2-nitro-1-(4-nitrophenyl) ethanol. (B) 2-nitro-1-(3-nitrophenyl) ethanol. (C) 2-nitro-1-(2-nitrophenyl) ethanol. (D) 2-nitro-1-(4-bromophenyl) ethanol. (E) 2-nitro-1-p-tolylethanol. (F) 2-nitro-1-phenylethanol.

| A | 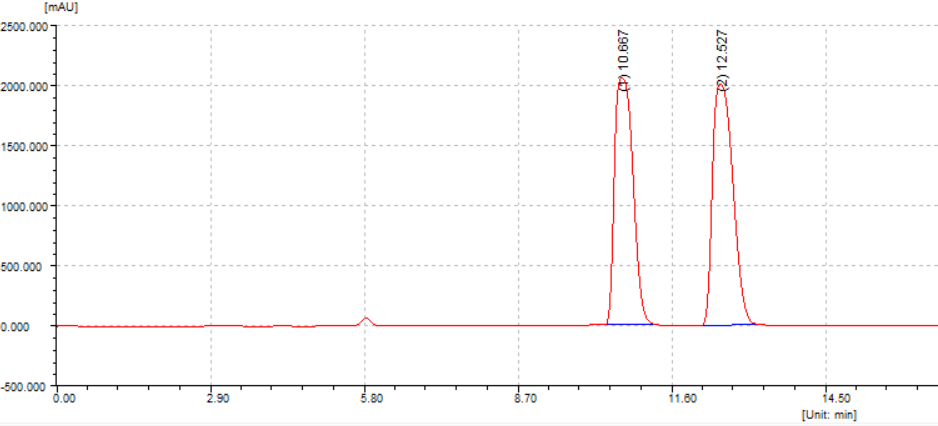 |
| --- | --- |
| B | 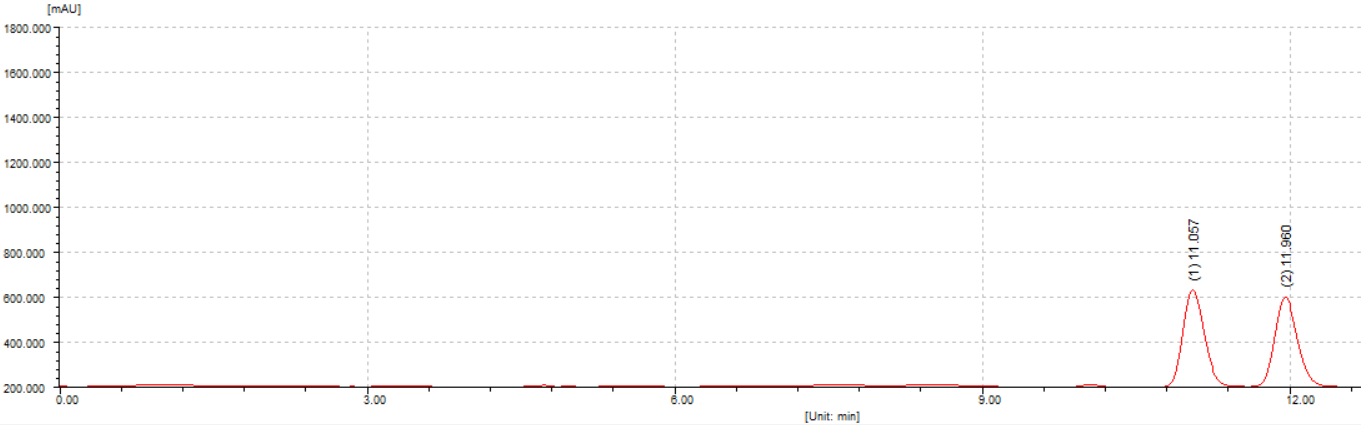 |
| C | 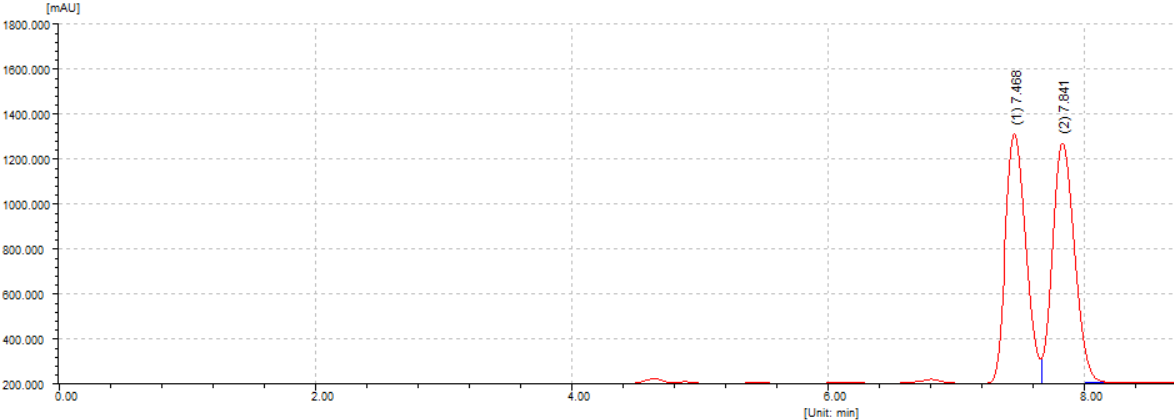 |
| D | 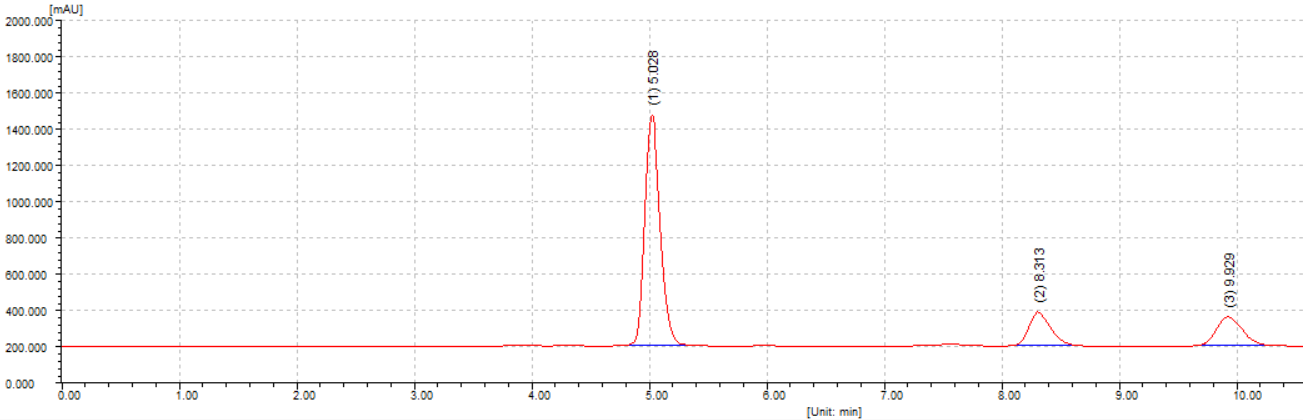 |
| E | 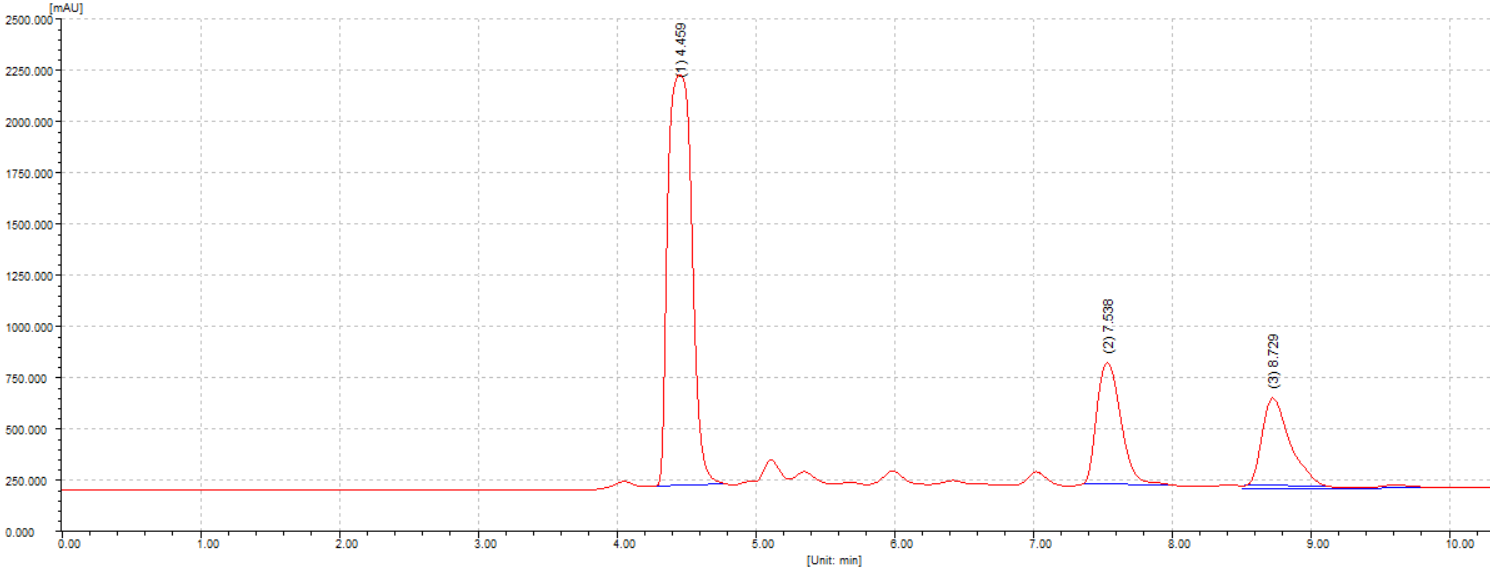 |
| F | 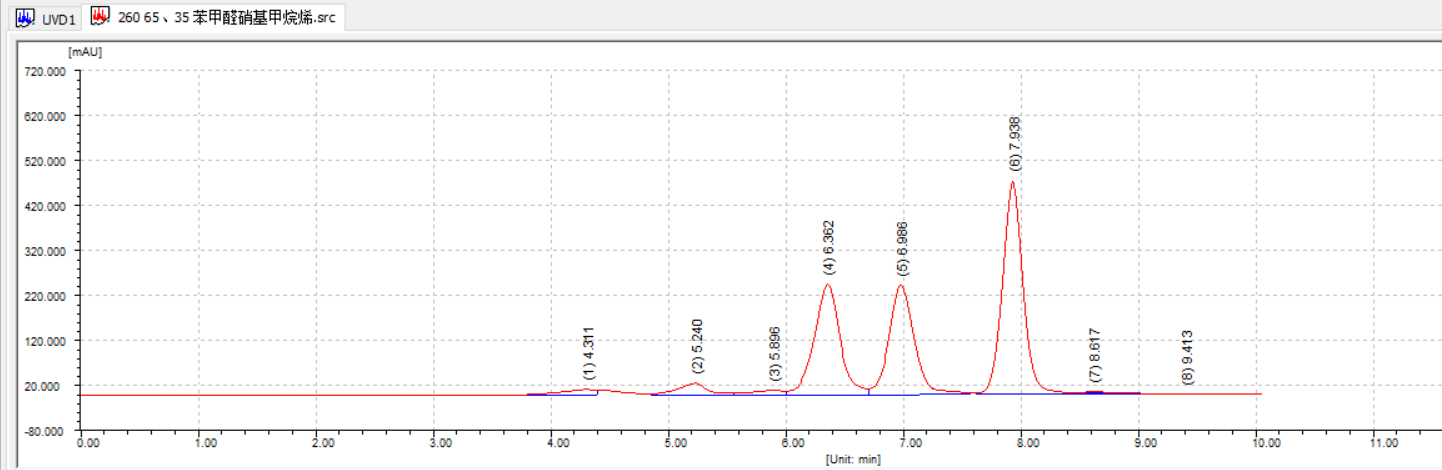 |

## Fig. S2 Liquid chromatography spectrums of β-nitroalcohols. (A)2-nitro-1-(4-nitrophenyl) ethanol. (B) 2-nitro-1-(3-nitrophenyl) ethanol. (C) 2-nitro-1-(2-nitrophenyl) ethanol. (D) 2-nitro-1-(4-bromophenyl) ethanol. (E) 2-nitro-1-p-tolylethanol. (F) 2-nitro-1-phenylethanol.
